# Predicting the primary infection source of *Escherichia coli* bacteremia using virulence-associated genes

**DOI:** 10.1007/s10096-024-04754-6

**Published:** 2024-01-25

**Authors:** Christian Schaadt Ilsby, Frederik Boetius Hertz, Henrik Westh, Jonathan Monk, Peder Worning, Helle Krogh Johansen, Katrine Hartung Hansen, Mette Pinholt

**Affiliations:** 1 Department of Clinical Microbiology, Copenhagen University Hospital Hvidovre, Hvidovre, Denmark; 2 Department of Clinical Microbiology, Copenhagen University Hospital Rigshospitalet, Copenhagen, Denmark; 3 Department of Immunology & Microbiology, University of Copenhagen, Copenhagen, Denmark; 4 Department of Clinical Medicine, University of Copenhagen, Copenhagen, Denmark; 5 Department of Bioengineering, University of California, San Diego, CA USA

**Keywords:** *E. coli*, WGS, Virulence-associated genes, Bacteremia, Urosepsis

## Abstract

**Purpose:**

To investigate the role of *E. coli* virulence-associated genes (VAGs) in predicting urinary tract infection (UTI) as the source of bacteremia in two distinct hospital populations, one with a large general catchment area and one dominated by referrals.

**Methods:**

*E. coli* bacteremias identified at Department of Clinical Microbiology (DCM), Hvidovre Hospital and DCM, Rigshospitalet in the Capital Region of Denmark from October to December 2018. Using whole genome sequencing (WGS), we identified 358 VAGs from 224 *E. coli* bacteremia. For predictive analysis, VAGs were paired with clinical source of UTI from local bacteremia databases.

**Results:**

VAGs strongly predicting of UTI as primary infection source of bacteremia were primarily found within the *pap* gene family. *papX* (PPV 96%, sensitivity 54%) and *papGII* (PPV 93%, sensitivity 56%) were found highly predictive, but showed low sensitivities. The strength of VAG predictions of UTI as source varied significantly between the two hospital populations. VAGs had weaker predictions in the tertiary referral center (Rigshospitalet), a disparity likely stemming from differences in patient population and department specialization.

**Conclusion:**

WGS data was used to predict the primary source of *E. coli* bacteremia and is an attempt on a new and different type of infection source identification. Genomic data showed potential to be utilized to predict the primary source of infection; however, discrepancy between the best performing profile of VAGs between acute care hospitals and tertiary hospitals makes it difficult to implement in clinical practice.

**Supplementary Information:**

The online version contains supplementary material available at 10.1007/s10096-024-04754-6.

## Introduction

*Escherichia coli* is a commensal in the human gut. Certain strains of *E. coli* can cause infections in humans, including urinary tract infections (UTI), intraabdominal infections, and bacteremia. *E. coli* is the leading cause of bacteremia, with a 30-day mortality rate of nearly 10% [1–4]. In high-income countries, more than half of *E. coli* bacteremias originate from the urogenital tract [5].

UTI is a common infection defined as the presence of typical symptoms from the urinary tract and bacteriuria (presence of a significant amount of uropathogenic bacteria in urine) [6]. UTIs caused by *E. coli* account for approximately 75% of all UTIs [7]. Important risk factors for community-acquired UTI include female sex, age, immunosuppression, diabetes, urological abnormalities, and a history of previous UTIs [8, 9].

Identification of the primary infection source of *E. coli* bacteremia is crucial for various reasons. Most importantly, determining the source can guide more precise and effective treatment strategies like targeted antimicrobial therapy, length of treatment, and/or surgical interventions. Misidentifying or delaying the identification of the primary infection source could increase the risk of complications and mortality [10–12].

Whole genome sequencing (WGS) has revolutionized the study of bacterial infections, providing a comprehensive picture of the genetic makeup of a bacterial species. Virulence-associated genes (VAGs) are genes associated with bacterial pathogenesis, and their identification is crucial in understanding the mechanisms used by bacteria [13].

We aim to assess if information on specific bacterial VAGs could help to predict the source of infection of *E. coli* bacteremias and improve treatment decisions. The study will contribute to a better understanding of which VAGs play a part in bacteremia and can become an important tool for the clinicians to identify the primary source of infection in *E. coli* bacteremia.

## Materials and methods

### Isolate collection

The study was conducted in the Capital Region of Denmark at the Department of Clinical Microbiology (DCM) at Hvidovre Hospital (DCM-1) and Rigshospitalet (DCM-2). *E. coli* bacteremias identified from October to December 2018 were consecutively included in the study. Only monomicrobial bacteremias were included and only one positive culture was included per patient.

Blood culture bottles were incubated in the Bactec blood culture system (Bactec, BD Diagnostics, NJ, USA). Identification of bacterial species was done using matrix-assisted laser desorption/ionization time-of-flight mass spectrometry (MALDI-TOF MS) analysis (Bruker Daltonics, Germany). Included blood culture isolates were stored at − 80 °C.

### Bacteremia database and hospital descriptions

Local bacteremia databases stored data on acquisition (hospital- or community-acquired bacteremia), sex, and source of bacteremia. Clinical microbiology specialists or medical doctors in clinical microbiology training collected data prospectively for each bacteremia case. Primary bacteremia infection source was determined as UTI, non-UTI, or unknown source through medical journal reviews using clinical data, radiological data, and dialogue with clinicians. Patients with a positive blood culture taken within 48 h of admission were classified as community-acquired. Positive blood cultures taken later than 48 h of admission or blood cultures from patients readmitted within 48 h of discharge were classified as hospital-acquired.

The DCM-1 provides services for five secondary acute care referral hospitals, collectively offering over 1300 beds and serving a catchment area of approximately 1.1 million inhabitants. The hospitals covered by DCM-1 do not contain departments for hematology, oncology, or urology. Consequently, they do not specialize in the care of immunocompromised patients, transplant recipients, or patients undergoing or experiencing complications from urological surgery.

Rigshospitalet is the most specialized tertiary referral hospital in Denmark and contains departments that specialize in handling immunocompromised patients as they cover departments of hematology, oncology, rheumatology, neonatology, intensive care units, in addition to transplant patients. The DCM-2 only provides services for Rigshospitalet which has a total capacity of approximately 1100 beds and has no regular catchment area.

Due to the very different patient populations and medical specialties serviced by DCM-1 and DCM-2, we decided to analyze the two isolate populations separately. This decision mitigates potential confounders introduced by pooling data and allows for more precise, context-specific insights into the VAGs of *E. coli* bacteremia.

### Whole genome sequencing, virulence-associated genes, and bacterial typing

*E. coli* isolates were sequenced using short-read WGS on the Illumina system at the Department of Genomic Medicine and DCM-2. Genome libraries were prepared using the NexteraXT kit and were sequenced using an Illumina NextSeq. The raw output fastq files are stored on a High-Performance Computing (HPC) cluster service by the National Life Science Supercomputing Center – Computerome at DTU and UCPH. From here, the raw fastq files undergo a quality control and quality assurance test using fastqc. Fastqc checks for per base sequence, quality, per base GC content, N content, as well as the sequence length distribution, kmer content, and for overrepresented sequences. Per base sequence quality > 30 across > 140 bases of each read, quality scores > 36 for > 90% of reads, per base sequence content 25% for each base across positions 20–140 in each read, and a distribution of per sequence GC content with median of 52% and standard deviation of 5% as expected for *E. coli* strains. A strict cutoff for number of reads required to obtain > 50 × coverage was also enforced.

Paired-end reads were assembled using SPAdes (v3.11.0) annotated using PROKKA (v1.12) with the *Escherichia* genus setting [14, 15]. VAGs were identified by using BLASTp with the amino acid sequences of each translated open reading frames against the Virulence Factor Database (VFDB—2019) [16] and National Center for Biotechnology Information (NCBI, Bethesda, MD, USA—2023). A requirement of 80% sequence identity across at least 80% of the length of the protein sequence was enforced for positive hits of VAGs.

A system for naming the identified VAGs was imposed. Predominantly, VAGs were named according to the gene feature of the corresponding NCBI nucleotide page. As certain VAGs had no useful name findable, for ease of reading, these were dubbed an abbreviated form of their NCBI protein name and suffixed with roman numerals in case of duplicates. The self-named VAGs are kept non-cursive in the article. A list of VAGs along with VFID, NCBI accession numbers, and representative sequences are found in Table 4 in Supplementary Appendix.

To confirm that the *E. coli* population was a heterogeneous group and not belong to a clonal outbreak, relatedness of *E. coli* was determined by MLST and core genome MLST (cgMLST). MLST and cgMLST were performed using SeqSphere + v7.2.3 (Ridom GmbH, Munster, Germany). The maximum allelic distance allowed for two samples to be considered from the same cluster was set to 10 alleles according to the clustering rules from SeqSphere + (https://www.cgmlst.org/ncs/schema/Ecoli845/). Minimum spanning trees (MSTs) were constructed to visualize the genetic relatedness among the *E. coli* isolates.

### Data analysis

To determine specific *E. coli* VAGs and/or combinations of VAGs that would best predict a UTI as the primary source of the bacteremia, we compiled a list of the 358 VAGs containing the single VAGs and combined VAGs as both pairwise and triple-wise cross-pairings. The pairwise cross-pairing resulted in 63,903 pairs and the triple-wise cross-pairing resulted in 7,583,156 triplets. These VAGs or VAG combinations were then coupled with clinical data containing information on UTI status (UTI as the primary source of bacteremia or non-UTI source). We calculated the prevalence (the proportion of *E. coli* isolates with a given VAG or VAG combination), estimates for positive predictive value (PPV) (the proportion of patient isolates with a given VAG or VAG combination who had UTI as source), and sensitivity (the proportion of UTI source cases in the study population correctly detected with the specific VAG or combination of VAGs) (Table 5). A sorting was applied to exclude VAGs or VAG combinations having a prevalence of < 20%. The 20 VAG singles, pairs, and triplets with the highest PPVs were subjected to bootstrapping simulations of 100,000 repetitions within each DCM population. Afterwards, the high-performing 20 VAG singles, pairs, and triplets from each DCM population were tested out on the opposing DCM population with new test estimates and bootstrapping simulations calculated. No prevalence requirement was imposed here. 95% confidence intervals (CI) were calculated from nonparametric bootstrapping.

Finally, we aimed to examine to what extent our top-performing VAGs overlapped within isolate populations. To achieve this, we employed an iterative selection method to identify combinations of VAGs that would maximize the sensitivity estimate. This process involved examining all possible combinations of five individual VAGs identified in our high-PPV tables (Table 6, Table 7). The optimal combination and order were computed along with five sensitivity estimates for each combination. These sensitivity estimates were calculated as the proportion of bacteremia cases with UTI as source in which at least one of the VAGs in the combination is present. Maximizing sensitivity and minimizing isolate population overlapping allows us predictions for the vast majority of the bacteremia cases with UTI as source.

Statistical analysis and data handling were done using R (v. 4.2.2, R Foundation for Statistical Computing, Vienna, Austria).

## Results

**Fig. 1:**
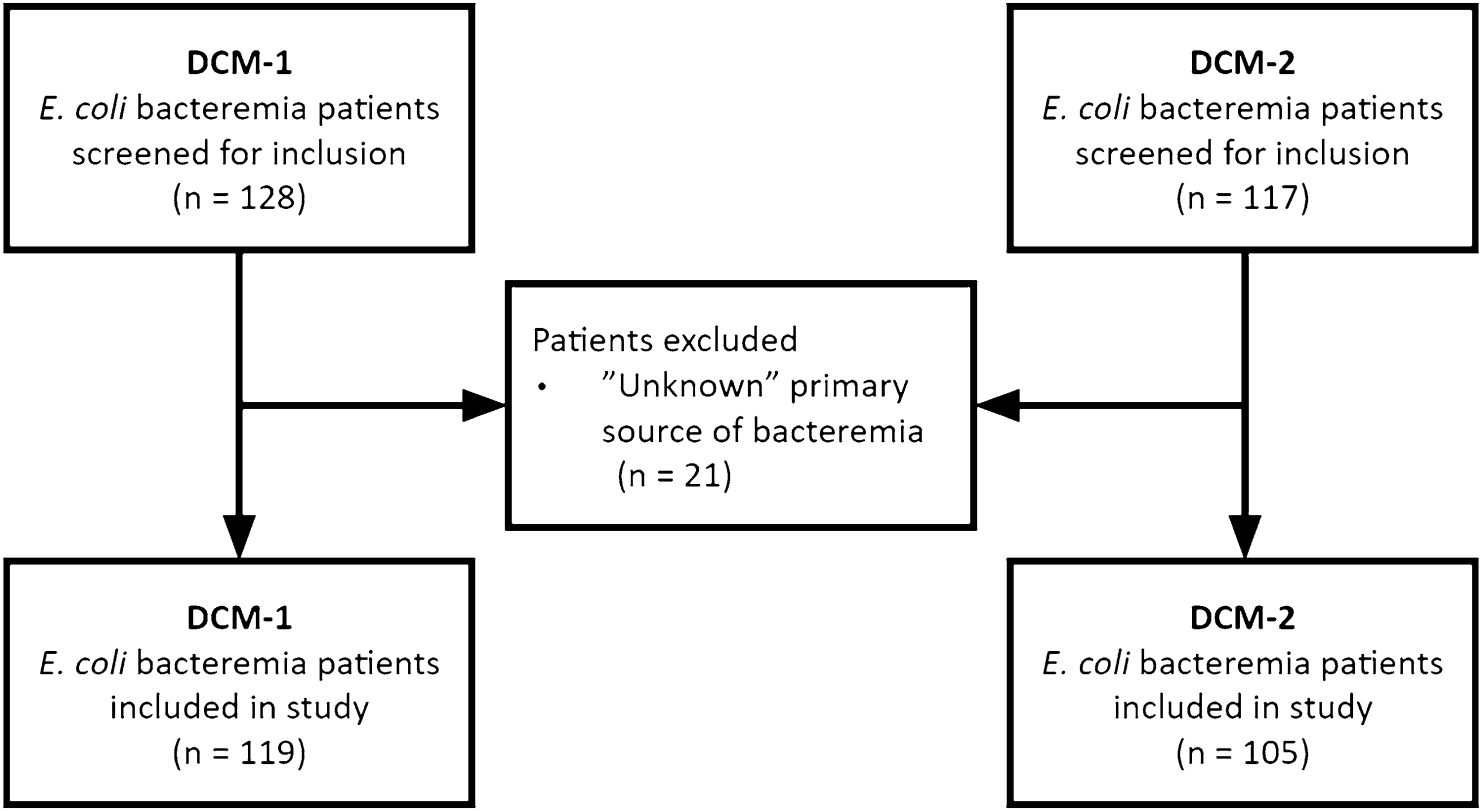
Flowchart of study patients from DCM-1 and DCM-2. Abbreviations: DCM, Department of Clinical Microbiology; WGS, whole genome sequencing

A total of 253 *E. coli* bacteremia cases were included in the study, 119 from DCM-1 and 105 from DCM-2. Twenty-nine cases were excluded due to unknown source of infection (*n* = 21) (Fig. 1). In total, there were 358 VAGs variably present across the assembled genomes.

Most cases at DCM-1 had UTI as source (81.51%), were female sex (58%), and were community-acquired (89.1%) (Table 1). This is consistent with the nature of the hospital as a secondary acute care referral hospital. Conversely, DCM-2, which contains tertiary referral hospital with specialized departments, showed an equal distribution between UTI and non-UTI as source of bacteremia (49.5% vs. 50.5%), fewer female cases (40%), and between community-acquired and hospital-acquired infections (48.6% vs. 51.4%) (Table 1). These differences likely reflect the complex patient population and specialized departments of DCM-2.

**Table 1:** Clinical characteristics of 224 *E. coli* bacteremia isolates from DCM-1 (*n* = 119) and DCM-2 (*n* = 105)

Category	DCM-1		DCM-2	
	*n*	%	*n*	%
Source of bacteremia				
UTI	97	81.5	52	49.5
Non-UTI	22	18.5	53	50.5
Acquisition				
Community-acquired	106	89.1	51	48.6
Hospital-acquired	13	10.9	54	51.4
Sex				
Female	69	58.0	42	40.0
Male	50	42.0	63	60.0

Examining the best performing single VAGs, VAG pairs, and VAG triplets in the DCM-1 bacteremia population, *papX* had the highest PPV of 96% (95% confidence interval (CI): [90, 100]) and a sensitivity of 54% (CI [44, 64]). *intS*, *papE*, *papD*, and *papGII* had PPVs of 93–95% and a sensitivity ranging from 34 to 56% (Table 2). Of note, *papX* was present in all top five-performing combinations. Multiple *pap* genes (*papC*, *papF*, *papH*) were present in the top five pairs, all predicting 100% PPV (CI [100, 100]) while still correctly predicting approximately half of the UTI source cases (sensitivity 41–46%). Adding triplets of VAGs did not improve the sensitivity.

**Table 2:** Displaying the five *E. coli* VAG singles, pairs, and triplets that best predict UTI as source of bacteremia from DCM-1 (*n* = 119) and DCM-2 (*n* = 105)

VAG combinations*			Prevalence, %^*1*^	PPV, % (CI)^*2*^	Sensitivity, % (CI)^*3*^
﻿DCM-1					
*papX*			45	96 (90–100)	54 (44–64)
*intS*			33	95 (87–100)	38 (29–48)
*papE*			30	94 (86–100)	35 (26–45)
*papD*			29	94 (85–100)	34 (25–44)
*papGII*			49	93 (86–98)	56 (46–66)
*ykfF*	*papX*		39	100 (100–100)	47 (38–57)
*papX*	*papC*		36	100 (100–100)	44 (34–54)
*papX*	*papF*		34	100 (100–100)	42 (33–52)
*papX*	*papH*		34	100 (100–100)	42 (33–52)
*papX*	*papI*		34	100 (100–100)	42 (33–52)
*papX*	*fecE*	*fepE*	34	100 (100–100)	42 (42–42)
*papX*	*fecA*	*fepE*	34	100 (100–100)	42 (42–42)
*papX*	*fecB*	*fepE*	34	100 (100–100)	42 (42–42)
*papX*	*fecC*	*fepE*	34	100 (100–100)	42 (42–42)
*papX*	*fecD*	*fepE*	34	100 (100–100)	42 (42–42)
DCM-2					
*kpsT*			23	67 (47–85)	31 (19–44)
*fimC4*			20	67 (45–86)	27 (15–40)
*fimD*			20	67 (45–86)	27 (15–40)
*fimA*			21	64 (43–83)	27 (15–40)
*vat*			26	63 (44–81)	33 (20–46)
*istA*	*insF*		20	76 (57–94)	31 (19–44)
*kpsM*	*insF*		21	73 (53–90)	31 (19–44)
*kpsT*	gtpp		21	73 (53–90)	31 (19–44)
*insF*	hypp		24	72 (53–89)	35 (22–48)
*ykfF*	*insF*		23	71 (52–88)	33 (20–46)
*kpsM*	*z1226*	*hemR*	26	78 (61–93)	40 (27–54)
*z1226*	is3-I	*chuY*	22	78 (60–95)	35 (22–48)
*z1226*	is3-I	*chuA*	22	78 (60–94)	35 (22–48)
*z1226*	is3-I	*chuT*	22	78 (60–94)	35 (22–48)
*z1226*	is3-I	*chuS*	22	78 (60–95)	35 (22–48)

*Virulence-associated gene descriptions and representative sequences are found in Table 4 in Supplementary Appendix

Sorted to display the highest PPV within each group and with a requirement of prevalence values > 20%. ^*1*^*Prevalence:* The proportion (%) of isolates with the specific VAG or VAG combination. ^*2*^*PPV:* The proportion (%) of cases with the specific VAG or VAG combination associated with UTI as source of bacteremia. ^*3*^*Sensitivity:* The proportion (%) of cases with UTI as source of bacteremia correctly predicted with the specific VAG or VAG combination. Abbreviations: *VAG*, virulence-associated gene; *CI*, confidence interval; *PPV*, positive predictive value; *DCM*, Department of Clinical Microbiology; *UTI*, urinary tract infection

In the DCM-2 bacteremia population, the best predicting single VAG was *kpsT* (PPV = 67%; CI [47, 85], sensitivity = 31%; CI [19, 44]) (Table 2). Adding pairs and triplets of VAGs increased the PPVs and sensitivity values noticeably compared to the DCM-1 population with the best ranked VAG triplet in the DCM-2 population being the combination of *kpsM*, z1226, and *hemR* (PPV = 78%; CI [61, 93], sensitivity = 40%; CI [27, 54]). No *pap* genes or other VAGs present in the top predicting VAGs from DCM-1 were present.

For additional single, pair, or triplet combinations with sensitivity estimates from each hospital, see Table 6 and Table 7 in the supplementary tables.

Results from the best predicting VAG combinations from the DCM-1 population (Table 2) run on the DCM-2 population data resulted in comparatively overall low sensitivity values and PPV values were also comparatively low except for *papD* with 71% (CI [47, 92]) and *papGII* with 68% (CI [46, 89]) (Table 8). Vice versa, results from the best predicting VAG combinations from the DCM-2 population (Table 2) run on DCM-1 population data performed had only VAG *kpsT* (PPV = 90%; CI [78, 100], sensitivity = 29%; CI [20, 38]) and in combination as *kpsT* and gtpp (PPV = 90%; CI [78, 100], sensitivity = 28%; CI [19, 37]) perform well with PPV value ≥ 90% (Table 8).

### Iterative sensitivity optimization

Using an iterative selection approach to optimize sensitivity population coverage and minimize isolate population overlapping, we identified the optimal combinations and order of VAGs to check for in series. The combinations with the largest sensitivity values for DCM-1 population were the VAG group of *papX*, *intS*, *kpsT*, *insN*, and *papC* (maximum sensitivity = 92.8%) (Table 3). For the DCM-2 population, VAG group of *kpsT*, *fimD*, *intS*, *hemR*, and is3-II was found optimal in terms of individual PPV values and UTI source case sensitivity coverage (maximum sensitivity = 84.6%) (refer to Table 6 and Table 7 for individual PPV values).

**Table 3:** Iterative sensitivity optimization showing top 5 VAGs with stepwise addition for maximized sensitivity in each DCM population

DCM-1				DCM-2			
VAGs*	*n*	Sensitivity (%)	PPV (%)	VAGs*	*n*	Sensitivity (%)	PPV (%)
*papX*	52	53.6	96.3	*kpsT*	16	30.8	67.8
*papX* or *intS*	62	63.9	93.4	*kpsT* or* fimD*	26	50.0	65.0
*papX* or *intS* or* kpsT*	69	71.1	92.0	*kpsT* or *fimD* or* intS*	34	65.4	61.8
*papX* or *intS* or *kpsT* or *insN*	81	83.5	89.0	*kpsT* or *fimD* or *intS* or *hemR*	39	75.0	57.4
*papX* or *intS* or *kpsT* or *insN* or* papC*	90	92.8	88.2	*kpsT* or *fimD* or *intS* or *hemR* or is3-II	44	84.6	53.0

*Virulence-associated gene descriptions and representative sequences are found in Table 4 in Supplementary Appendix

Derived from Table 6 and Table 7, these VAG sequences aim to maintain high PPV values while maximizing population sensitivity. Refer to Table 6 and Table 7 for individual PPV values of each single VAG. *N*, the number (*n*) of cases correctly predicted as UTI source out of respectively 97 for DCM-1 and 52 for DCM-2; *PPV*, the proportion (%) of cases with one of the listed VAGs on each line associated with UTI as source; *Sensitivity*, the proportion (%) of cases with UTI as source of bacteremia correctly predicted with one of the listed VAG on each line. Abbreviations: *VAG*, virulence-associated gene; *CI*, confidence interval; *PPV*, positive predictive value; *DCM*, Department of Clinical Microbiology; *UTI*, urinary tract infection

### Phylogenetic analyses

MLST and cgMLST analyses for the DCM-1 and DCM-2 populations revealed a diverse population structure which ensures that the study is performed on a diverse *E. coli* population and not on clonal isolates (Figs. 2 and 3). Among examined isolates, ST 131 was the most frequent, with 33 occurrences (Table 9).

## Discussion

We evaluated 358 VAGs from genomes of 224 *E. coli* bacteremias, exploring their predictive value for UTI as the infection source of bacteremia.

We found that several VAGs predicted UTI as source in *E. coli* bacteremias quite well based on high PPVs. PPVs for DCM-1 and DCM-2 were respectively 93–100% and 67–78%, however with low sensitivities (DCM-1 34–54% and DCM-2 27–40%). The sensitivity increased to 85–93% by applying an “and/or” logic to various VAG combinations. Various *pap* genes performed well in the DCM-1 patient population in terms of both PPV and the sensitivity values and pairing these did increase slightly the PPV and sensitivities (e.g., *papX*, *papC*). In the DCM-2 patient population, *kps* and *fim* genes provide among the highest PPV values. Sensitivity values and PPV values remained low across tables for VAGs found in the DCM-2 patient population. In addition, the best performing combinations of VAGs at DCM-1 did not predict well in DCM-2 data.

Most studies examining UTI-related VAGs or virulence factors (VFs) to date have focused on *E. coli* in urine samples with much fewer studies focusing on *E. coli* bacteremia [1]. Recently, Kim et al. [17] examined the genomic difference between bacteremic UTI and non-bacteremic UTI caused by *E. coli*. With a study population of 80 *E. coli* UTI patients, of these 40 urine sample isolates and 40 blood sample isolates, they found no VFs associated with bacteremia. In contrast, Denamur et al. [18] examined *E. coli* bacteremia isolates in a genome-wide association study and found several *pap* genes (most notably the *papGII* operon) highly associated to the urinary tract as portal of entry, which support our findings. In the same study, a putative integrase gene, *opgE*, was also described and found associated to UTI as source; however, this gene was not part of the database applied to identify VAGs.

In light of previous research, it appears that certain VAGs or VFs, particularly within the *pap* gene cluster, may be indicators for UTI as source of *E. coli* bacteremia. The *pap* genes are a class of VFs that play a significant role in the pathogenesis of UTI. The *pap* operon coding for P fimbria, a chaperone-usher pathway (CUP) pilus, is located on pathogenicity islands [19]. P fimbriae are involved in adhesion to host tissues, an important step in the establishment of infection [7, 20, 21]. VFs like *papC* and *papGII* are well described as essential components of P fimbria assembly unlike *papX* [22]. Despite being less studied in existing literature, *papX—*one of the VAGs we found highly predictive of UTI as source in our study—is thought to regulate bacterial motility and expression of other *E. coli* fimbriae [23].

Whereas VAGs *kpsT* and *kpsM* are scarcely described in literature, the *fim* genes have a well-established role in UTI pathogenesis [24–26]. *fim*-genes encode the type I fimbriae, another class of CUP pili. Like P fimbriae, type I fimbriae facilitate the adherence of *E. coli* to host tissues, enabling initial colonization and persistent infection. The fimbriae are assembled by a conserved chaperone-usher mechanism, with *fimH* acting as the adhesin and other *fim* genes such as *fimA*, *fimC*, and *fimD* contributing to the complex’s assembly and transport [21, 27, 28]. Our study identified several *fim*-related VAGs as having strong predictive value for UTI as source as single VAGs in the DCM-2 patient population and much less so the *pap* genes, suggesting differences related to either foci of infections or host susceptibility factors like immunological status between the two study populations.

We selected our study populations to deliberately stem from two very different hospital setups. Having a population comprised of primarily complicated UTIs and various degrees of compromised immune systems likely makes it difficult to accurately select which patients suffer from ordinary lower UTI or pyelonephritis. The *E. coli* strains found in bacteremias with suspected urogenital origin from severely immunocompromised patients could also differ from those patients with normal immune system function [29]. While hospital setups such as the service area for DCM-1 appear more suited for using VAG data to predict bacteremia source origin, our findings suggest this depends heavily on patient population with no straightforward way of generalizing between hospital setups.

Study limitations include the skewed proportion of UTI as source and non-UTI as source in the DCM-1 patient population as compared to the more evenly distributed DCM-2 patient population. Not providing a specificity value is due to the VAGs being selected based on a uropathogenic profile and as such the study is not set up for examining VAGs predicting against UTI as source. While certain VAGs and combinations of VAGs emerge as significant in our data, the exact functional roles or synergistic interactions between the VAGs that might explain their predictive superiority remain outside the scope of this study. Study strengths include our large study population subjected to WGS and clinical data from our bacteremia database.

Identification of the primary source of infection will improve treatment, reduce side effects, and reduce risks associated with diagnostic procedures. We attempted to predict infection source backwards from blood culture findings using data on our landscape of local bacterial genomics. VAGs could be identified by designing a multiplex PCR targeting a list of VAGs with high individual PPV values and in unison a high cumulative sensitivity value. Long-read sequencing on the Oxford Nanopore platform could also be used to provide clinicians with fast results regarding VAGs. However, discrepancies between hospital populations require each hospital to derive its own prediction profile of VAGs.

In conclusion, genomic data showed potential to be utilized to predict the primary source of infection in *E. coli* bacteremia, specifically in UTIs as source of origin. However, discrepancy between best performing profile of VAGs between acute care referral hospitals (DCM-1) and a tertiary hospital (DCM-2) makes it difficult to implement in clinical practice. Comparatively, the *pap* genes performed the best in our analysis. Within the DCM covering acute care referral hospitals, VAGs *papX* and *papGII* were found to be both moderately sensitive and highly predictive of UTI as source of infection for *E. coli* bacteremia. However, no single VAGs were useful by themselves as sequentially checking a group of VAGs seems a more practical approach. The effectiveness of VAGs in predicting bacteremia source seems also to depend strongly on hospital type and patient population with no reliable ability to transfer predictions between hospital types. Future studies can test the reported predictions on external datasets. Data in larger scale will hopefully provide us more knowledge.

### Supplementary Information

Below is the link to the electronic supplementary material.

Supplementary file1 (DOCX 371 KB)

## Data Availability

The datasets analyzed in the study are available at https://www.ncbi.nlm.nih.gov/bioproject/1037322.
