# Supplementary material for: Predicting the primary infection source of Escherichia coli bacteremia using virulence-associated genes
Source: Eur J Clin Microbiol Infect Dis. 2024 Jan 25;43(4):641–8. doi: 10.1007/s10096-024-04754-6 (PMC10965582; doi:10.1007/s10096-024-04754-6)
Supplement: Supplementary file 1 — Supplementary file1 (DOCX 371 KB) [file 10096_2024_4754_MOESM1_ESM.docx]

**Supplementary Appendix**

**Fig. 2 MST of *E. coli* isolates from DCM-1**


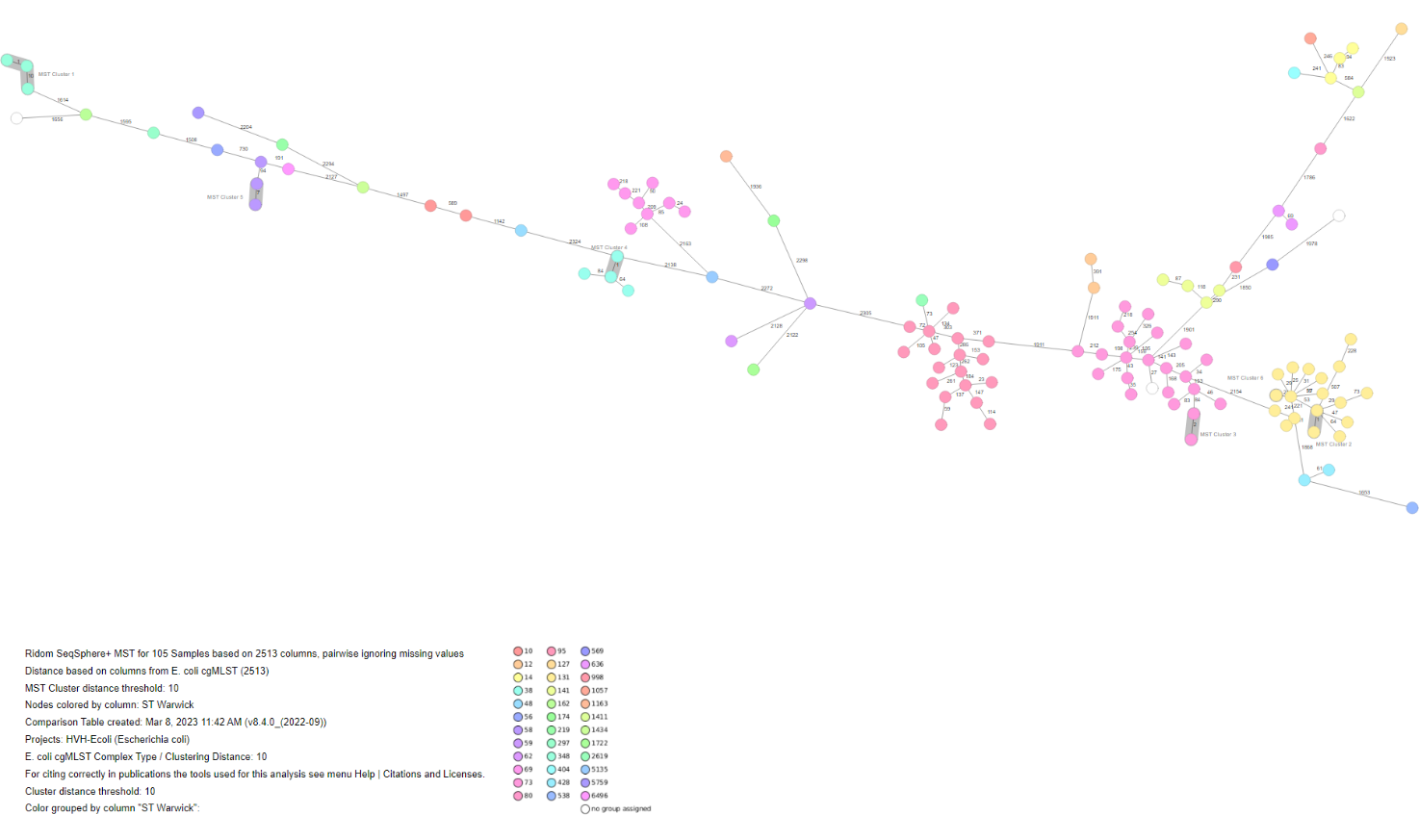


**Fig. 2**: Shows the minimum spanning tree (MST) of study patient *E. coli* isolates from DCM-1, colored by MLST and labelled by allele distances. Abbreviations: DCM, Department of Clinical Microbiology; MST, Minimum spanning tree; MLST, multilocus sequence typing.

**Fig. 3 MST of *E. coli* isolates from DCM-2**

**
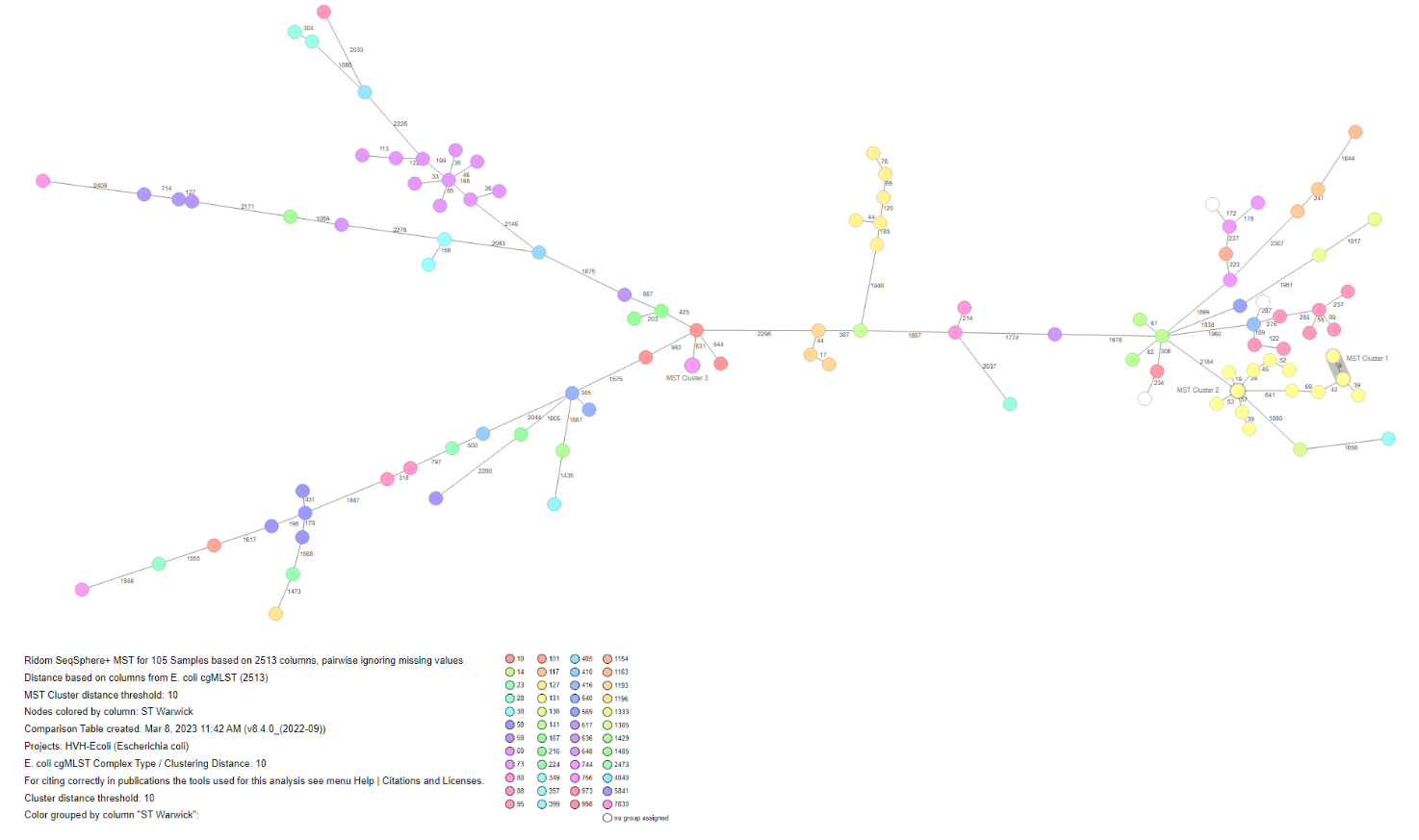
**

**Fig. 3**: Shows the minimum spanning tree (MST) of study patient *E. coli* isolates from DCM-2, colored by MLST and labelled by allele distances. Abbreviations: DCM, Department of Clinical Microbiology; MST, Minimum spanning tree; MLST, multilocus sequence typing.

**Table 4 Overview of VAGs presented in article**

| VAG | VFID | NCBI Accession | Representative Sequence |
| --- | --- | --- | --- |
| *papX* | VFG1541 | WP_250665357.1 | *MNNTDTLEKIIRHQKNKDPAYPFREHLLMQLCIRTNKRMQDNISEFLGVYGINHSVYMVLTTLFAAESHCLSPSEISQKLQFTRTNITRITDFLEKAGYVKRMDSREDRRAKKISLTSEGMFFIQRLTLAQSMYLKEIWGYLTHDEQELFEVINKKLLAHLDDVSS* |
| *intS* | VFG0626 | WP_001218804.1 | *MALTDAKIRAAKPTDKAYKLTDGAGMFLLVHPNGSRYWRLRYRILGKEKTLALGVYPEVSLSEARTKRDEARKLISEGIDPCEQKRVKKVVPDLQLSFEHIARRWHASNKQWAQSHSDKVLKSLETHVFPFIGNRDITTLNTPDLLIPVRAAEAKQIYEIASRLQQRISAVMRYAVQSGIIRYNPALDMAGALTTVKRQHRPALELSRLPELLSRIDGYKGQPVTRLAVMLNLLVFIRSSELRYARWSEIDIDNSMWTIPAEREPLPGVKFSHRGSKMRTPHLVPLSKQVVAILAELQTWAGENGLIFTGAHDPRKPISENTVNKALRVMGYDTTQEVCGHGFRAMACSALIESGLWSRDAVERQMSHQERNGVRAAYIHKAEHLEERRLMLQWWADFLDANREKGISPFEYAKINNPLK* |
| *papE* | VFG0888 | WP_000723799.1 | *MYRIFQMKKIRGLCLPVMLGAVLMSQHVHAADNLTFKGKLIIPACTVTKAEVDWGNVEIQTLSQNGNHEKEFTVNMQCPYHLGTMKVTITATNTYNNAILVQNTSNTSSDGVLVYLYNSNAGNIGTAITLGTPFTPGKITGNNADRTISLHAKLGYKGNMQSLKAGDFSATATLVASYS* |
| *papD* | VFG0885 | WP_235637098.1 | *MTLDISNDNKQLPYLAQAWIENENQEKIIAGPVIATPPVQRLEPGAKSMVRLSTTPDISKLPQDRESLFYFNLREIPPRSEKANVLQIALQTKIKLFYRPAAIKTRPNEVWQDQLILNKVSGGYRIENPTPYYVTVIGLGGSEKQAEEGEFETVMLSPRSEQTVKSANYNTPYLSYINDYGGRPVLSFICNGSRCSVKKEK* |
| *papGII* | VFG0890 | WP_000758683.1 | *MKKWFPALLFSLCVSGESSAWNNIVFYSLGNVNSYQGGNVVITQRPQFITSWRPGIATVTWNQCNGPEFADGSWAYYREYIAWVVFPKKVMTKNGYPLFIEVHNKGSWSEENTGDNDSYFFLKGYKWDERAFDAGNLCQKPGETTRLTEKFNDIIFKVALPADLPLGDYSVTIPYTSGIQRHFASYLGARFKIPYNVAKTLPRENEMLFLFKNIGGCRPSAQSLEIKHGDLSINSANNHYAAQTLSVSCDVPANIRFMLLRNTTPTYSHGKKFSVGLGHGWDSIVSVNGVDTGETTMRWYKAGTQNLTIGSRLYGESSKIQPGVLSGSATLLMILP* |
| *ykfF* | VFG1525 | WP_001278287.1 | *MSDCHPVLLPEGPFSREQAVAVTTAYRNVLIEDDQGTHFRLVIRNAEGQLRWRCWNFEPDAGKQLNSYLASEGILRQ* |
| *papC* | VFG0895 | WP_000654937.1 | *MNLVMRGMKDRIPFAVNNITCVILLSLFCNAASAVEFNTDVLDAADKKNIDFTRFSEAGYVLPGQYLLDVIVNGQSISPASLQISFVEPQSSGDKAEKKLPQACLTSDMVRLMGLTAESLDKVVYWHDGQCADFHGLPGVDIRPDTGAGVLRINMPQAWLEYSDATWLPPSRWDDGIPGLMLDYNLNGTVSRNYQGGDSHQFSYNGTVGGNLGPWRLRADYQGSQEQSRYNGEKTTNRNFTWSRFYLFRAIPRWRANLTLGENNINSDIFRSWSYTGASLESDDRMLPPRLRGYAPQITGIAETNARVVVSQQGRVLYDSMVPAGPFSIQDLDSSVRGRLDVEVIEQNGRKKTFQVDTASVPYLTRPGQVRYKLVSGRSRGYGHETEGPVFATGEASWGLSNQWSLYGGAVLAGDYNALAAGAGWDLGVPGTLSADITQSVARIEGERTFQGKSWRLSYSKRFDNADADITFAGYRFSERNYMTMEQYLNARYRNDYSSREKEMYTVTLNKNVADWNTSFNLQYSRQTYWDIRKTDYYTVSVNRYFNVFGLQGVAVGLSASRSKYLGRDNDSAYLRISVPLGTGTASYSGSMSNDRYVNMAGYTDMFNDGLDSYSLNAGLNSGGGLTSQRQINAYYSHRSPLANLSANIASLQKGYTSFGVSASGGATITGKGAALHAGGMSGGTRLLVDTDGVGGVPVDGGQVVTNRWGTGVVTDISSYYRNTTSVDLKRLPDDVEATRSVVESALTEGAIGYRKFSVLKGKRLFAILRLADGSQPPFGASVTSEKGRELGMVADEGLAWLSGVTPGETLSVNWDGKIQCQVNVPETAISDQQLLLPCTPQK* |
| *papF* | VFG0889 | WP_263866955.1 | *MFSVPLSGGGMARLSLFISLLLTSVAVLADVQINIRGNVYIPPCTINNGQNIVVDFGNINPEHVDNSRGEVTKTISISCTYKSGSPWIKVTGNAMAGQTNVLATNIANFGIALYQGKGMSTPLTLGNGSGNGYRVTAGLDTARSTFTFTSVPFRNGSRTLNGGDFRTTASMSMIYN* |
| *papH* | VFG0883 | WP_001239363.1 | *MRLRFSVPLFFFCCVFVHGVFAGPFPPPGMSLPEYWGEEHVWWDGRAAFHGEVVRPACTLAMEDAWQIIDMGETPVRDLQNGFSGPERKFSLRLRNCEFNSQGGNLFSDSRIRVTFDGVRGETPDKFNLSGQAKGINLQIADARGNIARAGKVMPAIPLTGNEEALDYTLRIVRNGKKLEAGNYFAVLGFRVDYE* |
| *papI* | VFG0880 | WP_000006206.1 | *MSEYMKNEILEFLNRHDGGKTAEIAEALAVTDYQARYYLLLLEKAGMVQRSPLRRGMATYWFLKGEKQAGQSCSSTT* |
| *fecE* | VFG1042 | WP_000175457.1 | *MTLRTENLTVSYGTDKVLNDVSLSLPTGKITALIGPNGCGKSTLLNCFSRLLMPQSGTVFLGDNPINMLSSRQLARRLSLLPQHHLTPEGITVQELVSYGRNPWLSLWGRLSAEDNARVNVAMNQTRINHLAVRRLTELSGGQRQRAFLAMVLAQNTPVVLLDEPTTYLDINHQVDLMRLMGELRTQGKTVVAVLHDLNQASRYCDQLVVMANGHVMAQGTPEEVMTPGLLRTVFSVEAEIHPEPVSGRPMCLMR* |
| *fepE* | VFG0927 | WP_000096764.1 | *MSSLNIKQGSEAHFPEYPLASPSNNEIDLLNLIEVLWRAKKTVMAVVFAFACAGLLISFILPQKWTSAAVVTPPEPVQWQELEKTFTKLRVLDLDIKIDRTEAFNLFIKKFQSVSLLEEYLRSSPYVMDQLKEAKIDPLDLHRAIVALSEKMKAVDDNASKKKDESALYTSWTLSFTAPTSEEAQKVLAGYIDYISALVVKESIENVRNKLEIKTQFEKEKLAQDRIKTKNQLDANIQRLNYSLDIANAAGIKKPVYSNGQAVKDDPDFSISLGADGIERKLEIEKAVTDVAELNGELRNRQYLVEQLTKTNINDVNFTPFKYQLRPSLPVKKDGQGKAIIVILSALVGGMVACGGVLLRHAMASRKQDAMMADHLV* |
| *fecA* | VFG1047 | WP_141106350.1 | *MTPLRVFRKTTPLVNAIRLSLLPLAGLSFSAFAAQVNIAPGSLDKALNQYAAHSGFTLSVDASLTRGKQSNGLHGDYDVESGLQQLLDGSGLQVKPLGNNSWTLEPAPAPKEDALTVVGDWLGDARENDVFEHAGARDVIRREDFAKTGATTMREVLNRIPGVSAPENNGTGSHDLAMNFGIRGLSPRLASRSTVLMDGIPVPFAPYGQPQLSLAPISLGNMDAIDVVRGGGAVRYGPQSVGGVVNFVTRAIPQDFGIEAGVEGQLSPTSSQNNPKETHNLMVGGTADNGFGTALLYSGTRGSDWREHSATRIDDLMLKSKYAPDEVHTFNSLLQYYDGEADMPGGLSRADYDADRWQSTRPYDRFWGRRKLASLGYQFQPDSQHKFNIQGFYTQTLRSGYLEQGKRITLSPRNYWVRGIEPRYSQIL* |
| *fecB* | VFG1045 | WP_000879153.1 | *MLAFIRFLFAGLLLVISHAFAAMVQDEHGTFTLEKTPQRIVVLELSFADALAAVDVSPIGIADDNDAKRILPEVRAHLKPWQSVGTRAQPSLEAIAALKPDLIIADSSRHAGVYIALQQIAPVLLLKSRNETYAENLQSAAIIGEMVGKKREMQARLEQHKERMAQWASQLPKGTRVAFGTSREQQFNLHTQETWTGSVLASLGLNVPAAMAGSSMPSIGLEQLLAVNPAWLLVAHYREESIVKRWQQDPLWQMLTAAQKQQVASVDSNTWARMRGIFAAERIAADTVKIFHHQPLTVVK* |
| *fecC* | VFG1044 | WP_262825893.1 | *MLLWGLPVAALIIIFWLSLFCYSAIPVSGADATRALLPGHTPTLPEALVQNLRLPRSLVAVLIGASLALAGTLLQTLTHNPMASPSLLGINSGAALAMALTSALSPTPIAGYSLSFIAACGGGVSWLLVMTAGGGFRHTHDRNKLILAGIALSAFCMGLTRITLLLAEDHAYGIFYWLAGGVSHARWQDVWQLLPVVVTAVPVVLLLANQLNLLNLSDSTAHTLGVNLTRLRLVINMLVLLLVGACVSVAGPVAFIGLLVPHLARFWAGFDQRNVLPVSMLLGATLMLLADVLARALAFPGDLPAGAVLALIGSPCFVWLVRRRG* |
| *fecD* | VFG1043 | WP_000684856.1 | *MKIALVIFITLALAGCALLSLHMGVIPVPWRALLTDWQAGREHYYVLMEYRLPRLLLALFVGAALAVAGVLIQGIVRNPLASPDILGVNHAASLASVGALLLMPSLPVMVLPLLAFAGGMAGLILLKMLAKTHQPMKLALTGVALSACWASLTDYLMLSRPQDVNNALLWLTGSLWGRDWSFVKIAIPLMILFLPLSLSFCRDLDLLALGDARATTLGVSVPHTRFWALLLAVAMTSTGVAACGPISFIGLVVPHMMRSITGGRHRRLLPVSALTGALLLVVADLLARIIHPPLELPVGVLTAIIGAPWFVWLLVRMR* |
| *kpsT* | VFG1449 | WP_000590258.1 | *MIKIENLTKSYRTPTGRHYVFKNLNIIFPKGYNIALIGQNGAGKSTLLRIIGGIDRPDSGNIITEHKISWPVGLAGGFQGSLTGRENVKFVARLYAKRDELNERVDFVEEFSELGKYFDMPIKTYSSGMRSRLAFGLSMAFKFDYYLIDEITAVGDAKFKKKCSDIFDKIREKSHLIMVSHSERALKEYCDVAIYLNKEGQGKFYKNVTEAIADYKKDL* |
| *fimC4* | VFG1468 | WP_000943621.1 | *MLRFVLFFFTLSVSAVVQGSVVIMGTRVVYPATQKSISIRLNNDNESPALVQSWLDDGDAAAPPESVHVPFIITPPIFRMDSKSGQTVRIVYTGESLPKDRESLFYLNVLDIPAKPGAKKDSDNSEQRNNNYLQLAIRSRIKFFFRPAHLKLTPNDAYLKVTWHQEEGRIKAVNPTPYYITYNKIAVDQNKHLMPVEHGGMIPPFSSTVFALKGRTAPASKVSWVIVNDYGGYQQGESVLE* |
| *fimD* | VFG1467 | WP_011579244.1 | *MITEDISKENLFWSKVIMNANNLSCLIYCRCSLLLFAALGLTVTNHSFAAEEAEFDSEFLHLDKGINAIDIRRFSHGNPVPEGRYYSDIYVNNVWKGKADLQYLRTANTGAPTLCLTPELLSLIDLVKDTMSGNTSCFPASTGLSSASINFDLSTLRLNIEIPQALLNTRPRGYISPSQWQSGVPAAFINYDANYYQYSSSGTSNEQTYLGLKAGFNLWGWALRHRGSESWNNSYPAGYQNIETSIMHDLAPLRAQFTLGDFYTNGELMDSLSLRGVRLASDERMLPGSLRGYAPAVRGIANSNAKVTIYQNAHILYETTVPAGPFVINDLYPSGYAGDLIVKITESNGQTRMFTVPFAAVAQLIRPGFSRWQMSVGKYRYANKTYNDLIAQGTYQYGLTNDITLNSGLTTASGYTAGLAGLAFNTPLGAIASDITLSRTAFRYSGVTRKGYSLHSSYSINIPASNTNITLAAYRYSSKDFYHLKDALSANHNAFIDDVSVKSTAFYRPRNQFQISINQELGEKWGGMYLTGTTYNYWGHKGSRNEYQMGYSNFWKQLGYQIGLSQSRDNEQQRRDDRFYINFTLPLGESVQSPVFSTVLNYSKEEKNSIQTSISGTGGEDNQFSYGLSGNSQENGPSGYAMNGGYRSPYVNITTTVGHDTQNNNQRSFGASGAVVAHPYGVTLSNDLSDTFAIIHAEGAQGAAINNASGSRLDFWGNGIVPYVTPYEKNQISIDPSNLDLNVELSATEQEIIPRANSATLVKFDTKTGRSLLFDIRMSTGNPPPMASEVLDEHGQLAGYVAQAGKVFTRGLPEKGHLSVVWGPDNKDRCSFVYHVAHNKDDMQSQLVPVLCIQHPNQEKT* |
| *fimA* | VFG1466 | WP_000821052.1 | *MKRIFFIPLFLILLPKLAVAGPDDYVPSQIAVNTSTLPGVVIGPADAHTYPRVIGELAGTSNQYVFNGGAIALMRGKFTPALPKIGSITYTFHQGNSRDSSDFDIYDIGVSGLGIIIGMAGYWPATPLVPINSSGIYIDPVGANTNPNTYNGATASFGARLFVAFVATGRLPNGYITIPTRQLGTILLEAKRTSLNNKGLTAPVMLNGGRIQVQSQTCTMGQKNYVVPLNTVYQSQFTSLYKEIQGGKIDIHLQCPDGIDVYATLTDASQPVNRTDILTLSSESTAKGFGIRLYKDSDVTAISYGEDSPVKGNGSQWHFSDYRGEVNPHINLRANYIKIADATTPGSVKAIATITFSYQ* |
| *vat* | VFG0904 | WP_001034006.1 | *MNKIYALKYCYITNTVKVVSELARRVCKGSTRRGKRLSVLTSLALSALLPTVAGASTVGGNNPYQTYRDFAENKGQFQAGATNIPIFNNKGELVGHLDKAPMVDFSSVNVSSNPGVATLINPQYIASVKHNKGYQSVSFGDGQNSYHIVDRNEHSSSDLHTPRLDKLVTEVAPATVTSSSTADILNPSKYSAFYRAGSGSQYIQDSQGKRHWVTGGYGYLTGGILPTSFFYHGSDGIQLYMGGNIHDHSILPSFGEAGDSGSPLFGWNTAKGQWELVGVYSGVGGGTNLIYSLIPQSFLSQIYSEDNDAPVFFNASSGAPLQWKFDSSTGTGSLKQGSDEYAMHGQKGSDLNAGKNLTFLGHNGQIDLENSVTQGAGSLTFTDDYTVTTSNGSTWTGAGIIVDKDASVNWQVNGVKGDNLHKIGEGTLVVQGTGVNEGGLKVGDGTVVLNQQADSSGHVQAFSSVNIASGRPTVVLADNQQVNPDNISWGYRGGVLDVNGNDLTFHKLNAADYGATLGNSSDKTANITLDYQTRPADVKVNEWSSSNRGTVGSLYIYNNPYTHTVDYFILKTSSYGWFPTGQVSNEHWEYVGHDQNSAQALLANRINNKGYLYHGKLLGNINFSNKATPGTTGALVMDGSANMSGTFTQENGRLTIQGHPVIHASTSQSIANTVSSLGDNSVLTQPTSFTQDDWENRTFSFGSLVLKDTDFGLGRNATLNTTIQADNSSVTLGDSRVFIDKKDGQGTAFTLEEGTSVATKDADKSVFNGTVNLDNQSVLNINEIFNGGIQANNSTVNISSDSAVLENSTLTSTALNLNKGANVLASQSFVSDGPVNISDATLSLNSRPDEVSHTLLPVYDYAGSWNLKGDDARLNVGPYSMLSGNINVQDKGTVTLGGEGELSPDLTLQNQMLYSLFNGYRNTWSGSLNAPDATVSMTDTQWSMNGNSTAGNMKLNRTIVGFNGGTSSFTTLTTDNLDAVQSAFVMRTDLNKADKLVINKSATGHDNSIWVNFLKKPSDKDTLDIPLVSAPEATADNLFRASTRVVGFSDVTPTLSVRKEDGKKEWVLDGYQVARNDGQGKAAATFMHISYNNFITEVNNLNKRMGDLRDINGEAGTWVRLLNGSGSADGGFTDHYTLLQMGADRKHELGSMDLFTGVMATYTDTDASAGLYSGKTKSWGGGFYASGLFRSGAYFDLIAKYIHNENKYDLNFAGAGKQNFRSHSLYAGAEVGYRYHLTDTTFVEPQAELVWGRLQGQTFNWNDSGMDVSMRRNSVNPLVGRTGVVSGKTFSGKDWSLTARAGLHYEFDLTDSADVHLKDAAGEHQINGRKDGRMLYGVGLNARFGDNTRLGLEVERSAFGKYNTDDAINANIRYSF* |
| *istA* | VFG1514 | WP_001298025.1 | *MVTFETVMEIKILHKQGMSSRAIARELGISRNTVKRYLQAKSEPPKYTPRPAVASLLDEYRDYIRQRIADAHPYKIPATVIAREIRDQGYRGGMTILRAFIRSLSVPQEQEPAVRFETEPGRQMQVDWGTMRNGRSPLHVFVAVLGYSRMLYIEFTDNMRYDTLETCHRNAFRFFGGVPREVLYDNMKTVVLQRDAYQTGQHRFHPSLWQFGKEMGFSPRLCRPFRAQTKGKVERMVQYTRNSFYIPLMTRLRPMGITVDVETANRHGLRWLHDVANQRKHETIQARPCDRWLEEQQSMLALPPEKKEYDVHPSENLVNFDKHPLHHPLSIYDSFCRGVA* |
| *insF* | VFG0785 | WP_000336967.1 | *MLTQNGVPMSRYRAGRLMKYLNLSSCQPGKHQYKNARQEHTCLPNLLERQFAVPEPDRVWCGDITYIWAGNRWCYLAVVMDLFARRVIGWSLSANADTALISSALRMAYEVRGQPRDVMFHSDQGSQYTGLKYQQLLWRYRIKQSVSRRGNCWDNSPMERFFRSLKTEWVPTDGYTGKDVARQQISSYILNYYNSVRPHHYNGGLTPEESENRYHFYCKTVASIT* |
| *kpsM* | VFG1450 | WP_000124301.1 | *MARSGFEVQKVTVEALFLREIRTRFGKFRLGYLWAILEPSAHLLILLGILGYVMHRTMPDISFPVFLLNGLIPFFIFSSISKRSIGAIEANQGLFNYRPVKPIDTIIARALLETLIYVAVYILLMLIVWMTGEYFEITNFLQLVLTWSLLIILSCGVGLIFMVVGKTFPEMQKVLPILLKPLYFISCIMFPLHSIPKQYWSYLLWNPLVHVVELSREAVMPGYISEGVSLNYLAMFTLVTLFIGLALYRTREEAMLTS* |
| gtpp* | VFG1062 | WP_252813503.1 | *MNPSDAIEAIEKPLSSLPYSLSRHILEHLRKLTSHEPVIGIMGKSGAGKSSLCNALFQGEVTPVSDVHAGTREVQRFRLSGHGHSMVITDLPGVGESRDRDAEYEALYRDILPELDLVLWLIKADDRALSVDEYFWRHILHRGHQQVLFVVTQADKTEPCHEWDMAGIQPSPAQAQNIREKTEAVFRLFRPVHPVVAVSARTGWELDTLVSALMTALPDHAASPLMTRLQDELRTESVRGQAREQFTGAVDRIFDTAESVCVASVARTVLRAVRDTVVSVARAVWNWIFF* |
| hypp* | VFG1723 | WP_000266543.1 | *MWLLIFWLSSPFNVSSGDARQPTLAVHSQAFHTERACRDALRAIKKENVGEFSLRGVCVSDGNTKEEAESS* |
| *z1226* | VFG1533 | WP_001280468.1 | *MSDITISRPEVLNGHTDVICSTSIRHILAVRKSALLQIDTLIRQLAEISVLTESIGGKTALDWAMKQDFRCGCWLMEKPETAMKAITRNLDREIWRDLIQRSGMLSLMDAQARDTWYRSLEYDNFPEISEANILSTFEQLHQNKDEVFERGVINVFRGLSWNYKTNCPCKFGSKIIVNNLVRWDRWGFHLITGQQADRLADLERMLHLFSGKPIPDNRENITLRLDDHIQSVQGKECYEDEMFSIRYFKKGSAHITFKNQELVDRLNDIIARHYPGMLPSL* |
| *hemR* | VFG1659 | WP_001445605.1 | *MYMNVIRTVICTLIILPVGLQAATSHSSMVKDTITIVATGNQNTVFETPSMVSVVTNDTPWSQNAVTSAGMLKGVAGLSQTGAGRTNGQTFNLRGYDKSGVLVLVDGVRQLSDMAKSSGTYLDPALVKRIEVVRGPNSSLYGSGGLGGVVDFRTADAADFLPPGETNGLSLWGNIASGDHSTGSGLTWFGKTGKTDALLSVIMRKRGNIYQSDGEHAPNKEKPAALFAKGSVGITDSNKAGASLRLYRNNTTEPGNSTQTHGDSGLRDRKTVQNDVQFWYQYAPVDNSLINVKSTLYLSDITIKTNGHNKTAEWRNNRTSGVNVVNRSHTLIFPGAHQLSYGAEYYRQQQKPEGSATLYPEGNIDFTSLYFQDEMTMKSYPVNIIVGSRYDRYKSFNPRAGELKAERLSPRAAISVSPTDWLMMYGSISSAFRAPTMAEMYRDDVHFYRKGKPNYWVPNLNLKPENNITREIGAGIQLDGLLTDNDRLQLKGGYFGTDARNYIATRVDMKRMRSYSYNVSRARIWGWDMQGNYQSDYVDWMLSYNRTESMDASSREWLGSGNPDTLISDISIPVGHRGVYAGWRAELSASATHVKKGDPHQAGYTIHSFSLSYKPVSVKGFEASVTLDNAFNKLAMNGKGVPLSGRTVSLYTRYQW* |
| is3-I* | VFG1631 | WP_128969725.1 | *MKYVFIENHRAEFSIKAMCRVLRVARSGWYVWLRRRHQMSLRQQFRLTCDAAVHKAFFEAKQRYGAPRLADEMPEFNIKTIAASLRRQGLRAKASRKFSPVSYRAHGLPVLENLLEQDFSASGPNQKWAGDITYLRTDEGWLYLAVVIDLWSRAVIGWSMSPRMTAQLACDALQMALWRRRRPESVIVHTDRGGQYCSGDYQALLKRHNLRGSMSAKGYCYDNACVESFFHSLKVECIHGERFSSREIMRATVFNYIECDYNRWRRHSACGGLSPEQFENHNLA* |
| *chuY* | VFG0921 | WP_000189356.1 | *MTPWLLFGAGGKGVGARTLELALAEQRPVVAVIRHADAATKLAQQGVQVFTGDACDASVVAAACRAAGPDALIISTMGGAQDYLAHRTVIDEAEKAGITRMILVTSLGCGDSWPFLSERAKAAFGQAVREKTLAESWLQTSQLDYAILRPGGLLDGAATGKAQRIQNQECHGFINRADVAAHIHELANAPALNQQVYSLIEPDLKPA* |
| *chuA* | VFG0917 | WP_195806574.1 | *MLALAVSATLPTFAFATETMTVTATGNARSSFEAPMMVSVIDTSAPENQTATSATDLLRHVPGITLDGTGRTNGQDVNMRGYDHRGVLVLVDGVRQGTDTGHLNGTFLDPALIKRVEIVRGPSALLYGSGALGGVISYDTVDAKDLLQEGQSSGFRVFGTGGTGDHSLGLGASAFGRTENLDGIVAWSSRDRGDLRQSNGETAPNDESINNMLAKGTWQIDSAQSLSGLVRYYNNDAREPKNPQTVEASDSSNPMVDRSTIQRDAQLSYKLAPQGNDWLNADAKIYWSEVRINAQNTGSSGEYREQITKGARLENRSTLFADSFASHLLTYGGEYYRQEQHPGGATTGFPQAKIDFSSGWLQDEITLRDLPITLLGGTRYDSYRGSSDGYKDVDADKWSSRAGMTINPTNWLMLFGSYAQAFRAPTMGEMYNDSKHFSIGRFYTNYWVPNPNLRPETNETQEYGFGLRFDDLMLSNDALEFKASYFDTKAKDYISTTVDFAAATTMSYNVPNAKIWGWDVMTKYTTDLFSLDVAYNRTRGKDTDTGEYISSINPDTVTSTLNIPIAHSGFSVGWVGTFADRSTHISSSYSKQPGYGVNDFYVSYQGQQALKGMTTTLVLGNAFDKEYWSPQGIPQDGRNGKIFVSYQW* |
| *chuT* | VFG0918 | WP_001081821.1 | *MNRRLYFIYNSNDNHDHSQFDKSSHIMPRIITRPFLFSPLTLCISAVVSAAKTMVKRKKLFTALLALSWTFSVTAAERIVVAGGSLTELIYAMGAGKRVVGVDETTSYPPETAKLPHIGYWKQLSSEGILSLRPDSVITWQDAGPQIVLDQLRAQKVNVVTLPRVPATLEQMYANIRQLAKTLQVPEQGEALVTQINQRLERVQQNVAAKKAPVKAMFILSAGGSAPQVAGKGSVADAILSLAGAENVATHQQYKSYSAESLIAANPEVIVVTSQMVDGDINRLRSIAGITHTAAWKNQRIITVDQNLILGMGPRIADVVESLHQQLWPQ* |
| *chuS* | VFG0916 | WP_001017192.1 | *MNHYTRWLELKEQNPGKYARDIAGLMNISEAELAFARVTHDAWRMRGDIREILAALESVGETKCICRNEYAVHEQVGAFTNQHLNGHAGLILNPRALDLRLFLNQWASVFHIKENTARGERQSIQFFDHQGDALLKVYATDNTDMAAWSELLARFITDENTPLELKAVDAPVVQTRADASVVEQEWRAMTDVHQFFTLLKRHNLTRQQAFNLVADDLACKVSNSALAQILESAQQDGNEIMVFVGNRGCVQIFTGVVEKVVPMKGWLNIFNPTFTLHLLEESIAEAWVTRKPTSDGHVTSLELFAHDGTQIAQLYGQRTEGEQEQAQWRKQIASLIPEGVTA* |
| *chuW* | VFG0919 | WP_001095619.1 | *MNTNNTLDLTPHFALDGDQPFKDRRAMMPFRGAIPVAKEQLAQTWQEMINQTVSPRKRLVYLHIPFCATHCTFCGFYQNRFNDDACAHYTDALIREIEMEADSVLHQSAPIHAVYFGGGTPSALSAHDLARIITTLREKLPLAPDCEITIEGRVLNFDAERIDACLDAGANRFSIGIQSFNSKIRKKMARTSDGPTAIAFMESLVKRDRAAVVCDLLFGLPGQDAQTWGEDLAIARDIGLDGVDLYALNVLPNTPLGKAVENGRTTVPSPAERRDLYLQGCDFMDDAGWRCISNSHWGRTTRERNLYNLLIKQGADCLAFGSGAGGSINGYSWMNERNLQTWHESVAAGKKPLMMIMRNAERNAQWRHTLQSGIETACVPLDELTPHAEKLAPLLAQWHQKGLSRDASTCLRLTNEGRFWASNILQSLNELIQVLNAPAIALEKP* |
| *insN* | VFG1485 | WP_001406079.1 | *MKKRNFSAEFKRESAQLVVDQNYTVADAAKAMDIGLSTMTRWVKQLRDERQGKTPKASPITPEQIEIRELRKKLQRIEMENEILKKATALLMSDSLNSSR* |
| *sat* | VFG0902 | WP_001034089.1 | *MREYMNKIYSLKYSAATGGLIAVSELAKRVSGKTNRKLVATMLSLAVAGTVNAANIDISNVWARDYLDLAQNKGIFQPGATDVTITLKNGDKFSFHNLSIPDFSGAAASGAATAIGGSYSVTVAHNKKNPQAAETQVYAQSSYRVVDRRNSNDFEIQRLNKFVVETVGATPAETNPTTYSDALERYGIVTSDGSKKIIGFRAGSGGTSFINGESKISTNSAYSHDLLSASLFEVTQWDSYGMMIYKNDKTFRNLEIFGDSGSGAYLYDNKLEKWVLVGTTHGIASVNGDQLTWITKYNDKLVSELKDTYSHKINLNGNNVTIKNTDITLHQNNADTTGTQEKITKDKDIVFTNGGDVLFKDNLDFGSGGIIFDEGHEYNINGQGFTFKGAGIDIGKESIVNWNALYSSDDVLHKIGPGTLNVQKKQGANIKIGEGNVILNEEGTFNNIYLASGNGKVILNKDNSLGNDQYAGIFFTKRGGTLDLNGHNQTFTRIAATDDGTTITNSDTTKEAVLAINNEDSYIYHGNINGNIKLTHNINSQDKKTNAKLILDGSVNTKNDVEVSNASLTMQGHATEHAIFRSSANHCSLVFLCGTDWVTVLKETESSYNKKFNSDYKSNNQQTSFDQPDWKTGVFKFDTLHLNNADFSISRNANVEGNISANKSAITIGDKNVYIDNLAGKNITNNGFDFKQTISTNLSIGETKFTGGITAHNSQIAIGDQAVVTLNGATFLDNTPISIDKGAKVIAQNSMFTTKGIDISGELTMMGIPEQNSKTVTPGLHYAADGFRLSGGNANFIARNMASVTGNIYADDAATITLGQPETETPTISSAYQAWAETLLYGFDTAYRGAITAPKATVSMNNAIWHLNSQSSINRLETKDSMVRFTGDNGKFTTLTVNNLTIDDSAFVLRANLAQADQLVVNKSLSGKNNLLLVDFIEKNGNSNGLNIDLVSAPKGTAVDVFKATTRSIGFSDVTPVIEQKNDTDKATWTLIGYKSVANADAAKKATLLMSGGYKAFLAEVNNLNKRMGDLRDINGESGAWARIISGTGSAGGGFSDNYTHVQVGADNKHELDGLDLFTGVTMTYTDSHAGSDAFSGETKSVGAGLYASAMFESGAYIDLIGKYVHHDNEYTATFAGLGTRDYSSHSWYAGAEVGYRYHVTDSAWIEPQAELVYGAVSGKQFSWKDQGMNLTMKDKDFNPLIGRTGVDVGKSFSGKDWKVTARAGLGYQFDLFANGETVLRDASGEKRIKGEKDGRMLMNVGLNAEIRDNLRFGLEFEKSAFGKYNVDNAINANFRYSF* |
| is3-II* | VFG1732 | WP_113708405.1 | *MDSARALIARGWGVSLVSRCLRVSRAQLHVILRRTDDWMDGRRSRHTDDTDVLLRIYHVIGELPTYGYRRVWALLRRQAELDGMPAINAKRVYRIMRQNALLLERKTAVPPSKRAHTGKVAVKESNQRWCSDGFEFRCDNGEKLRVTFALDCCDREALHWAVTTGGFDSETVQDVMLGAVERRFGNELPASPVEWLTDNGSCYRANETRQFARMLGLEPKNTAVRSPESNGIAESFVKTIKRDYISIIPKPDGLTAAKNLAEAFEHYNEWHPHSALGYRSPREYLRQRACNGLSDNRCLEI* |
| *iha* | VFG1731 | WP_001223352.1 | *MRITTLASVVIPCLGFSASSIAAAEDVMIVSASGYEKKLTNAAASVSVISQEELQSSQYHDLAEALRSVEGVDVESGTGKTGGLEISIRGMPASYTLILIDGVRQGGSSDVTPNGFSAMNTGFMPPLAAIERIEVIRGPMSTLYGSDAMGGVVNIITRKNADKWLSSVNAGLNLQESNKWGNSSQFNFWSSGPLVDDSVSLQVRGSTQQRQGSSVTSLSDTAATRIPYPTESQNYNLGARLDWKASEQDVLWFDMDTTRQRYDNRDGQLGSLTGGYDRTLRYERNKISAGYDHTFTFGTWKSYLNWNETENKGRELVRSVLKRDKWGLAGQPRELKESNLILNSLLLTPLGESHLVTVGGEFQSSSMKDGVVLASTGETFRQKSWSVFAEDEWHLTDALALTAGSRYEHHEQFGGHFSPRAYLVWDVADAWTLKGGVTTGYKAPRMGQLHKGISGVSGQGKTNLLGNPNLKPEESVSYEAGVYYDNPAGLNANVTGFMTDFSNKIVSYSINDNTNSYVNSGKARLHGVEFAGTLPLWSEDVTLSLNYTWTRSEQRDGDNKGAPLSYTPEHMVNAKLNWQITEEVASWLGARYRGKTPRFTQNYSSLSAVQKKVYDEKGEYLKAWTVVDAGLSWKMTDALTLNAAVNNLLNKDYSDVSLYSAGKSTLYAGDYFQTGSSTTGYVIPERNYWMSLNYQF* |
| is3-III* | VFG1706 | WP_050491594.1 | *MCQVFGVSRSGYYNWVQHEPSDRKQSDERLKLEIKVAHIRTRETYGTRRLQTELAENGIIVGRDRLARLRKELRLRCKQKRKFRATTNSNHNLPVAPNLLNQTFAPTAPNQVWVADLTYVATQEGWLYLAGIKDVYTCEIVGYAMGERMTKELTGKALFMALRSQRPPAGLIHHSDRGSQYCAYDYRVIQEQFGLKTSMSRKGNCYDNAPMESFWGTLKNESLSHYRFNNRDEAISVIREYIEIFYNRQRRHSRLGNISPATFREKYHQMAA* |
| *tnpB* | VFG1698 | WP_146715896.1 | *MIPLPSGTKIWLVAGITDMRNGFNGLAAKVQTTLKDDPMSGHVFIFRGRNGSQVKLLWSTGDGLCLLTKRLERGRFAWPSARDGKVFLTPAQLAMLLEGIDWRQPKRLLTSLTML* |
| is3-IV* | VFG0605 | WP_005099850.1 | *MLREGIRVARCTVARLMAVMGLAGVLRGKKVRTTISRKAVAAGDRVNRQFVAERPDQLWVADFTYVSTWRGFVYVAFIIDVFAGYIVGWRVSSSMETTFVLDALEQALWARRPSGTVHHSDKGSQYVSLVYTQRLKEAGLLASTGSTGDSYDNAMAESINGLYKAEVIHRKSWKNRAEVELATLTWVDWYNNRRLLERLGHIPPAEAEKAYYASIGNDDLAA* |
| *iucC* | VFG0617 | WP_001015713.1 | *MNHKDWDFVNRRLVAKMLSEMEYEQVFHAESQGDDHYCINLPGAQWRFIAERGIWGWLWIDAQTLRCTDEPVLAQTLLMQLKPVLSMSDATVAEHMQDLYATLLGDLQLLKARRGLSASDLINLDADRLQCLLSGHPKFVFNKGRRGWGKEALERYAPEYTNTFRLHWLAVKREHMIWRCDNDLDIQQLLTAAMDPQEFTRFSQVWQENGLDHNWLPLPVHPWQWQQKIATDFIADFAEGRMVSLGEFGDQWLAQQSLRTLTNASRRGGLDIKLPLTIYNTSCYRGIPGRYIAAGPLASRWLQQVFATDATLVQSGAVILGEPAAGYVSHEGYAALARAPYRYQEMLGVIWRENPCRWLKPDESPVLMATLMECDENNQPLAGAYIDRSGLDAETWLTQLFRVVVVPLYHLLCRYGVALIAHGQNITLAMKEGVPQRVLLKDFQGDMRLVKEAFPEMDSLPQEVRDVTSRLSADYLIHDLQTGHFVTVLRFISPLMVRLGVPERRFYQLLAAVLSDYMNKHPQMAERFALFSLFRPQIIRVVLNPVKLTWPDLDGGSRMLPNYLENLQNPLWLVTQEYES* |
| *tnpJ* | VFG1707 | NP_706716.1 | *MSRKNQRYSKEFKAEAVRTVLENQLSISEGASRLSLPEGTLGQWVTAARKGLGTPGSRTVAELESEILQLRKALNEARLERDILKKATAYFAQESLKNTR* |
| isl3* | VFG1718 | WP_139309522.1 | *MISDRDGRALALTDDCGTESLAGYLRTLTDEQLLAIKTLSMDMNAGYIRAARIHLPCAVEKIAFDRFHVAKQLGEVVDKTRQNEHPHLPVESWRQAKGARFLWQYSDKWMTKSRQEKLIWLRAQMKLTSQCWALKELAKDIWNRPWSEERRNDWERWLALAANSDVPMMKNAAKTIGKRLYGILNAMCLKRKRGGA* |
| *iucB* | VFG0616 | WP_001287501.1 | *MSEANIIHSRYGLRCEKLDKPLNLGWGLDNSAVLHCPGELPTGWLCDALDQIFIAAPQLSAVALPWAEWREEPQALTLFGQVKSDIIHRTAFWQLPLWLSSPANRASGEMVFDAEREIYFPQRPPRPQGEVYRRYDPRIRRMLSFRIADPVSDAERFTRWMNDPRVEYFWEQSGSLEVQTAYLERQLTGKHAFPLIGCFDDRPFSYFEIYWAAEDRIGRHYSWQPFDRGLHLLVGEQQWRGAHYVQSWLRGLTHYLLLDEPRTQRTVLEPRTDNQRLFRHLEPAGYRTIKEFDFPHKRSRMVMADRHHFFTEVGL* |
| *senB* | VFG1827 | WP_140426502.1 | *MLISLFLPTMAMAIDPPERELSRFALKTNYLQSPDEGVYELAFDNASKKVFAAVTDRVNREANKGYLYSFNSDSLKVENKYTMPYRAFSLAINQDKHQLYIGHTQSASLRISMFDTPTGKLVRTSDRLSFKAANAADSRFEHLRHMVYSQDSDTLFVSYSNMLKTAEGMKPLHKLLMLDGTTLALKGEVKDAYKGTAYGLTMDEKTQKIYVGGRDYINEIDAKNQTLLRTIPLKDPRPQITSVQNLAVDSASDRAFVVVFDHDDRSGTKDGLYIFDLRDGKQLGYVHTGAGANAVKYNPKYNELYVTNFTSGTISVVDATKYSITREFNMPVYPNQMVLSDDMDTLYIGIKEGFNRDWDPDVFVEGAKERILSIDLKKS* |
| *tnaC* | VFG0615 | WP_011069570.1 | *MLIMRIIIDIIVIILLCGSCLTMTLPSEKPATDVAAQCFLNALIRETTDWKLTEYPPDELLIPLDEQKSLHFRVAYFSPTQHHRFAFPARLVTASGSYPVDFTTLSRLIIDKLRHQLFLPVPLCETFHQRVLESHVHTQQAIDARHDWAALREKALNFGEAEQALLTGHAFHPAPKSHEPFNRREAERYLPDMAPHFPLRWFSVDKTQIAGESLHLNLQQRLTRFAAENAPQLLNELSDNQWLFPLHPWQGEYLLQQGWCQALVAKGLIKDLGEAGTSWLPTTSSRSLYCATSRDMIKFSLSVRLTNSIRTLSVKEVKRGMRLARLAQTDGWQMLQVRFPTFRVMQEDGWAGLLDLNGNIMQESLFALRENLLVDQPKSQTNVLVSLTQAAPDGGDSLLVSAVKRLSDRLGITVQQAAHAWVDAYCQQVLKPLFTAEADYGLVLLAHQQNILVQMLGDLPVGFIYRDCQGSAFMPHATDWLDSIGEAQAENIFTHEQLLRYFPYYLLVNSTFAVTAALGAAGLDSESNLMARVRASLAEVRDQVTHKTCLNYVLESPYWNVKGNFFCYLNDHNENTIVDPSVIYFDFANPLQAQEV* |
| *hlyA* | VFG0906 | WP_001142375.1 | *MPTITTAQIKSTLQSAKQSSANKLHSAGQSTKDALKKAAEQTRNAGNRLILLIPKDYKGQGSSLNDLVRTADELGIEVQYDEKNGTAITKQVFGTAEKLIGLTERGVTIFAPQLDKLLQKYQKAGNKLGGSAENIGDNLGKAGSVLSTFQNFLGTALSSMKIDELIKRQKSGSNVSSSELAKASIELINQLVDTAASINNNVNSFSQQLNKLGSVLSNTKHLTGVGNKLQNLPNLDNIGAGLDTVSGILSAISASFILSNADADTGTKAAAGVELTTKVLGNVGKGISQYIIAQRAAQGLSTSAAAAGLIASVVTLAISPLSFLSIADKFKRANKIEEYSQRFKKLGYDGDSLLAAFHKETGAIDASLTTISTVLASVSSGISAAATTSLVGAPVSALVGAVTGIISGILEASKQAMFEHVASKMADVIAEWEKKHGKNYFENGYDARHAAFLEDNFKILSQYNKEYSVERSVLITQQHWDTLIGELAGVTRNGDKTLSGKSYIDYYEEGKRLEKKPDEFQKQVFDPLKGNIDLSDSKSSTLLKFVTPLLTPGEEIRERRQSGKYEYITELLVKGVDKWTVKGVQDKGSVYDYSNLIQHASVGNNQYREIRIESHLGDGDDKVFLSAGSANIYAGKGHDVVYYDKTDTGYLTIDGTKATEAGNYTVTRVLGGDVKILQEVVKEQEVSVGKRTEKTQYRSYEFTHINGKNLTETDNLYSVEELIGTTRADKFFGSKFTDIFHGADGDDHIEGNDGNDRLYGDKGNDTLRGGNGDDQLYGGDGNDKLIGGTGNNYLNGGDGDDELQVQGNSLAKNVLSGGKGNDKLYGSEGADLLDGGEGNDLLKGGYGNDIYRYLSGYGHHIIDDDGGKDDKLSLADIDFRDVAFRREGNDLIMYKAEGNVLSIGHKNGITFRNWFEKESGDISNHQIEQIFDKDGRVITPDSLKKALEYQQSNNKASYVYGNDALAYGSQDNLNPLINEISKIISAAGNFDVKEERAAASLLQLSGNASDFSYGRNSITLTASA* |
| *insA* | VFG1690 | WP_001313605.1 | *MATVTVHRPRCNSDKVYRHGRSCSQHERFRCRSCKRVFQLTYSYEARKPGFKELIVEMAHNGTGAVISPEH* |
| is4-I* | VFG1657 | WP_033808980.1 | *MQTRDNLERMVVIKAFIAVRVLGLRQEGISEETQNDSCKKILTPTEWKLLWVKLEGKQLPSQTPTLKWACLKLGRWHDSKRTGRPGWVVMWDGWFRLQDMVEGYPVMKSLDQEI* |
| int-p4* | VFG1693 | WP_001218870.1 | *MALTDAKIRAAKPTDKAYKLTDGAGMFLLVHPNGSRYWRLRYRILGKEKTLALGVYPEVSLSEARTKRDEARKLISEGVDPCEQKRAKKVVPDLQLSFEHIARRWHASNKQWAQSHSDKVLKSLETHVFPFIGNRDITTLSTPDLLIPVRAAEVKQIYEIASRLQQRISAVMRYAVQSGIIRYNPALDMAGALTTVKRQHRPALDLSRLPELLSRINSYKGQPVTRLAVMLNLLVFIRSSELRYARWSEIDIDNAMWTIPAERKPLPGVKFSHRGSKMRTPHLVPLSQQAVAILAELQTWAGENGLIFTGAHDPRKPISENTVNKALRVMGYDTTKEVCGHGFRAMACSALIESGLWSRDAVERQMSHQERNGVRAAYIHKAEHLEERRLMLQWWADFLDANREECISPFEYAKVNNPLKR* |
| shiA-h* | VFG1694 | WP_000147023.1 | *MTGKLRFEVNDNQGCFIFPETWFGSLLDEFEELIDAYDADEISETSYINKLRRLARQENDFIDVHAYLAYVFLEQNAPRKALNAALKGLAVGNRLIPEGFSGRIIWIHPDNRPFLRALYAAILANAHLQRHQDAIMLIEKILDYNPEDNHGARWLLGPELLRTGAHEQARHILQEHADEFSPYWYELGLLHFLNGELVKAATAFRRGFAANTYIAEILCGNLHPFPLAVWHNFSGGPDTAEDYYATYHPLWGQYPEALLFVNWLYNHSSVLHERAEIIKCAEMLMQEDDFEICESILRQQEKLRERIDETLSEKIVQKCRNMNGEYVWPWILPFSAAGMKHTGIQYQ* |
| marR* | VFG1688 | WP_252350498.1 | *MSFLLPCGGIMNNIDLLKTITNYKKIKNPAYPAQESLLIHLYIRVNDKIQSIIENELSEYRINTSTFMVLVSLYMSDDYCQSPSDIYKELQFSKTNITHIIDKLEKKNIAKRINNKNDRRSKSICLTPDGVTLAQKLINTQNVMLKKIWSGLSDDEMKTFELANKKLLSNLNVN* |
| *papK* | VFG0887 | WP_000597713.1 | *MMIKSTGALLLFAALSAGQAIASDVAFRGNLLDRPCHVSGDSLNKHVVFKTRASRDFWYPPGRSPTESFVIRLENCHATAVGKIVTLTFKGTEEAALPGHLKVTGVNAGRLGIALLDTDGSSLLKPGTSHNKGQGEKVTGNSLELPFGAYVVATPEALRTKSVVPGDYEATATFELTYR* |
| *rhuM* | VFG0566 | WP_000774139.1 | *MADKYLTQSPAGEFVMFASDDGEVRVECRFEQETLWLPQATIANLYQITPQAVTQHIKAIYEEGELEQNATCKSYLQVQQEGSRQVSRNRLHYSLPVILAVGYRVRSPRGTQFRQWATQTLQKYLIKGFVMDDERLKNPPVGSSAVPDYFDEMLERIRDIRASERRVYLRVREIFALAADYQPSLKETTQFFQTIQNKLHFACTGHTAAELIHQRADASQPHMGLTSYKGEEVRKDDVTVAKNYLTQDEVSELNRVVNMWLDFAEDQARRRQQIFLRDWQDKLDQFLQFNDREVLQGAGKVTKKMADEKAQAEYSQFAEQQRRLKEAEGEKDIAGLLQWETEPKK* |
| *papJ* | VFG1546 | WP_000261304.1 | *MVVNKTTAVLYLIALSLSGFIHTFLRAEERGIYDDVFTADELRHYRINERGGRTGSLAISGALLSSPCTLVSNEVPLSLRPENHSAAAGAPLMLRLAGCGDGGALQPGKRGVAMTVSGSLVTGPGSGSALLPDRKLSGCDHLVIHDGDTFLLCRPDRRQEEMLAAWRKRATQEGEYSDARSNPAMLRLSIKYE* |
| *z1216* | VFG1527 | WP_000206652.1 | *MTTFSHSSTTPSVSATTASGNNQSQLVATPVPDEQRISFWPQHFGLIPQWVTLEPRVFGWMDRLCEDYCGGIWNLYTLNNGGAFIAPEPDEDDGETWILFNAMNGNRAEMSPEAAGIAACLMTYSHHACRTECYAMTVHYYRLRDYALQHPECSAIMRIID* |
| *iucA* | VFG0940 | WP_011069567.1 | *MIMRIIIDIIVIILLCGSCLTMTLPSEKPATDVAAQCFLNALIRETTDWKLTEYPPDELIIPLDEQKSLHFRVAYFSPTQHHRFAFPARLVTASGSYPVDFTTLSRLIIDKLRHQLFLPVPLCETFHQRVLESHAHTQQAIDARHDWTALREKALNFGEAEQALLTGHAFHPAPKSHEPFNRREAERYLPDMAPHFPLRWFSVDKTQIAGESLHLNLQQRLTRFAAENAPQLLNELSDNQWLFPLHPWQGEYLLQQGWCQALVAKGLIKDLGEAGTSWLPTTSSRSLYCATSRDMIKFSLSVRLTNSIRTLSVKEVKRGMRLARLAQTDGWQMLQVRFPTFRVMQEDGWAGLLDLNGNIMQESLFALRENLLVDQPKSQTNVLVSLTQAAPDGGDSLLVSAVKRLSDRLGITVQQAAHAWVDAYCQQVLKPLFTAEADYGLVLLAHQQNILVQMLGDLPVGFIYRDCQGSAFMPHATDWLDSIGEAQAENIFTHEQLLRYFPYYLLVNSTFAVTAALGAAGLDSESNLMARVRASLAEVRDQVTHKTCLNYVLESPYWNVKGNFFCYLNDHNENTIVDPSVIYFDFANPLQAQEV* |
| *yeeV* | VFG0786 | WP_000854712.1 | *MKTLPDTHVREASRCPSPITIWQTLLGRLLDQHYGLTLNDTPFADERVIEQHIEAGISLCDAVNFLVEKYVLVRTDQPGFSACTRSQLINSIDILRARRATGLMTRDNYRTVNNITLGKHPEAK* |
| *ag43* | VFG1063 | WP_000820563.1 | *MLNTSYRLVWNHITGTLVVASELARSRGKRAGVAVVLSLAAVTSIPALAADTIEQAGETVNGGTLTNHDNQIVLGTANGMTISSGLEYGPDNEANTGGQWIQNGGIANNTTVTGGGLQRVNAGGSVSDTVISAGGGQSLQGQAVNTTLNGGEQWVHEGGIATGTVINEKGWQTVKSGAMATDTVVNTGAEGGPDAENGDTGQFVRGNAVRTTINENGRQIVAAEGTANTTVVYAGGDQTVHGHALDTTLNGGYQYVHNGGTASGTVVNSDGWQIIKEGGLADFTTVNQKGKLQVNAGGTATHVTLKQGGALVTSTAATVLGSNRLGNFTVENGKADGVVLESGGRLDVLEGHSAQKTRVDDGGTLAVSAGGKATGVTMTSGGALIADSGATVEGTNASGKFSIDGTSGQASGLLLENGGSFTVNAGGLASNTTVGHRGTLTLAAGGSLSGRTQLSKGASMVLNGDVVSTGDIVNAGEIRFDNQTTPDAALSRAVAKGDSPVTFHKLTTSNLTGQGGTINMRVRLDGSNTSDQLVINGGQATGKTWLAFTNVGNSNLGVATSGQGIRVVDAQNGATTKEGAFALSRPLQAGAFNYTLNRDSDEDWYLRSENAYRAEVPLYTSMLTQAMDYDRILAGSRSHQTGVNGENNSVRLSIQDGHLGHDNNGGIARGATPESSGSYGFVRLEGDLLRTEVAGMSLTTGVYGAAGHSSVDVKNDDGSRAGTVRDDAGSLGGYLNLTHTSSGLWADIVAQGTRHSMKASSDNNDFRARGWGWLGSLETGLPFSITDNLMLEPQLHYTWQGLTLDDGQDNAGYVKFGHGSAQHVRAGFRLGSHNDMSFGEGTSSRDTLRDSTKHRVSELPVNWWVQPSVIRTFSSRGDMSMGTAAAGSNMTFSPSRNGTSLDLQAGLEARVRENITLGVQAGYAHSVSGSSAEGYNGQATLNVTF* |
| *fimB* | VFG0871 | WP_000790574.1 | *MKNKADNKKRNFLTHSEIESLLKAANTGPHAARNYCLTLLCFIHGFRASEICRLRISDIDLKAKCIYIHRLKKGFSTTHPLLNKEIQALKNWLSIRTSYPHAESEWVFLSRKGNPLSRQQFYHIISTSGGNAGLSLEIHPHMLRHSCGFALANMGIDTRLIQDYLGHRNIRHTVWYTASNAGRFYGIWDRARGRQRHAVL* |
| *kpsD* | VFG1448 | WP_001331697.1 | *MKLFKSILLIAACHAAQASAAIDINADPNLTGAAPLTGILNGQQSDTQNMSGFDNTPPPSPPVVMSRMFGAQLFNGTSADSGATVGFNPDYILNPGDSIQVRLWGAFTFDGALQVDPKGNIFLPNVGPVKVAGVSNSQLNALVTSKVKEVYQSNVNVYASLLQAQPVKVYVTGFVRNPGLYGGVTSDSLLNYLIKAGGVDPERGSYVDIVVKRGNRVRSNVNLYDFLLNGKLGLSQFADGDTIIVGPRQHTFSVQGDVFNSYDFEFRESSIPVTEALSWARPKPGATHITIMRKQGLQKRSEYYPISSAPGRMLQNGDTLIVSTDRYAGTIQVRVEGAHSGEHAMVLPYGSTMRAVLEKVRPNSMSQMNAVQLYRPSVAQRQKEMLNLSLQKLEEASLSAQSSTKEEASLRMQEAQLISRFVAKARTVVPKGEVILNESNIDSVLLEDGDVINIPEKTSLVMVHGEVLFPNAVSWQKGMTTEDYIEKCGGLTQKSGNARIIVIRQNGAAVNAEDVDSLKPGDEIMVLPKYESKNIEVTRGISTILYQLAVGAKVILSL* |
| *rox* | VFG0651 | WP_001095918.1 | *MNTPVSLMDDQMVDMSFITQLTGLTDKWFYKLIKHGAFPPPIKLGRSSRWLKSEVEAWLQARIAQSRP* |
| trpos* | VFG0603 | WP_000165821.1 | *MTKPVSISKKPRKQHTPEFRNEALKLAERIGVAAAARELSLYESQLYAWRSKQQQQMSSSERESELAAENVRLKRQLAEQAEELSILQKAATYFAKRLK* |
| *z1203* | VFG1504 | WP_000199856.1 | *MTSDFSAEKILAGKNVLYAAIHRIEWLFETFSSVCLSFSGGKDSTVLLHLAADVARRKKRRFSVLFIDWEAQYQCTIEHVQKMREMYRDVTDTFYWVALPLTTVNGVSQFQPEWICWEPDVTWVRHPPEYAITDMAYFPFYRYAMTFEEFVPAFSSWFAGNRCGVAILTGVRADESLNRFMGLVSQRKLRYADDKPWTTASPEGFYYTMYPLYDWKARDIWIYNARTRAIYNPLYDLMYRAGVPLRNMRVCEPFGPEQRKGLWLYHVLEPETWARMCERVSGAASGALYANESGAYFALRKRITKPPHHTWRSYAMFLLDVMPERTAEHYRNKIAVYLRWYQTRGFPDDIPDEQENDLGSRDIPSWRRICKTLIKNDFWCRTLSFSPNKPRHYERYLQRMKERRKEWGIL* |
| *z1204* | VFG1505 | WP_000502866.1 | *MGDSVTPEVEVLSNMIRQYFSQERSEEETIRALNHLRRVLHEVSPFAQEPVDCVLWVKADEVVANDYNPNVMSSSEKKLLKHSLEQDGFTQPVVVSEEKEHYLVVDGFHRQLLGRKADTRKRLKGWLPVACINPERKGQASRIAATIRHNRARGKHQITSMSDIVRDLSRLGWTDERIGTELGMDQDEVLRLKQISGLTELFQEENFSPAWTVR* |
| is4-II* | VFG1655 | WP_052985761.1 | *MQLSRLTLRSKKPELVEQELWGVLLAYNLVRYQMIKMAEHLKGYWPNQLSFSESCGMVMRMLMTLQGASPGRIPELMRDLASMGQLVKLPTRRERAFPRVVKERPWKYPTAPKKSQSVA* |
| *chuU* | VFG0922 | WP_000910402.1 | *MLKDSLSSARVFMGLSLLLLALVLFGASQGALKISFDALFDEEYRDIWLNIRLPRVLLAVLVGAALATAGVIMQGLFRNPMADPGLLGVSSGSALMVGVAIVLPFSFPVVLVLYEQMVFAIAGSLVVCTIIFLITQRHRDGSMMQLLLAGIAINALCGAAIGILSYIGDEQQLRQLTLWMMGNLGQAQWPTLLVASSFILPAIIATTCLAGTLNLLQLGDEEAHYLGVNVKRKRQQLLLVSSLLVGAAVSVSGIIGFIGLVIPHLIRMTTGANHRWLIPCSALAGACLLLMADTLARTLVQPAEMPVGLLTSLLGGPYFMWLILRNRRIT* |
| *fecI* | VFG1049 | WP_001283626.1 | *MSDRATTTASLTFESLYGTHHGWLKSWLTRKLQSAFDADDIAQDTFLRVMVSETLSTIRDPRSFLCTIAKRVMVDLFRRNALEKAYLEMLALMPEGGAPSPEERESQLETLQLLDSMLDGLNGKTREAFLLSQLDGLTYSEIAHKLGVSISSVKKYVAKAVEHCLLFRLEYGL* |
| *fecR* | VFG1048 | WP_262035786.1 | *MNPLLTDSRRQALRSASHWYAVLSGERVSPQQEARWQQWYEQDQDNQWAWQQVENLRNQLGGVPGDVASRALHDTRLTRRHVMKGLLLLLGAGGGWQLWQSETGEGLRADYRTAKGTVSRQQLEDGSLLTLNTQSAADVRFDAHQRTVRLWYGEIAITTAKDALQRPFRVLTRQGQLTALGTEFTVRQQDNFTQLDVQQHAVEVLLASAPAQKRIVNAGESLQFSASEFGAVKPLDDESTSWTKDILSFSDKPLGEVIATLTRYRNGVLRCDPAVAGLRLSGTFPLKNTDAILNVIAQTLPVKIQSITRYWINISPL* |

Table 4: VAGs presented in article tables (and supplementary tables) with VFID numbers, NCBI accession numbers and representative sequences. Abbreviations: VFID, virulence factor ID; NCBI, The National Center for Biotechnology Information; VAG, virulence associated gene.

*VAGs marked with (*) and non-cursive have been dubbed shortened names for ease of reading by article authors.

**Table 5 Calculation examples**

|  | | | DCM-1 | | |
| --- | --- | --- | --- | --- | --- |
|  | | | UTI source isolates* (n) | Non-UTI source isolates** (n) | Total isolates  (n) |
| Isolates **positive** for both *papX* and *papC**** | | | 43 | 0 | 43 |
| Isolates **negative** for both *papX* and *papC* | | | 54 | 22 | 76 |
| Total isolates (n) | | | 97 | 22 | 119 |
| Term | Definition | Calculation and example | | | |
| PPV | The proportion (%) of isolates with the specific gene combination associated with UTI as source of bacteremia. | $PPV=\frac{UTI source isolates positive for papX\mathrm{and}papC}{Total isolates positive for papX\mathrm{and}papC}=\frac{43}{43}=1 or 100 \%$ | | | |
| Sensitivity | The proportion (%) of cases with UTI as source of bacteremia correctly predicted with the specific gene combination. | $Sensitivity=\frac{UTI source isolates positive for papX\mathrm{and}papC}{Total UTI source isolates}=\frac{43}{97}=0.44 or 44 \%$ | | | |
| Prevalence | The proportion (%) of the total number of isolates where the specific gene combination is present. | $Prevalence=\frac{Total isolates positive for papX\mathrm{and}papC}{Total isolates}=\frac{43}{119}=0.36 or 36 \%$ | | | |

Table 5: Overview, calculation examples and definitions of PPV, sensitivity and prevalence for the pairwise VAG combination of *papX* and *papC* within the DCM-1 population. Abbreviations: PPV, positive predictive value; DCM, Department of Clinical Microbiology; UTI, urinary tract infection.

* “UTI source isolates” refers to *E. coli* bacteremia isolates with a UTI as source of infection.

** “Non-UTI source isolates” refers to *E. coli* bacteremia isolates with a cause other than UTI as source of infection.

*** Virulence associated gene descriptions and representative sequences are found in Table 4 in Supplementary Appendix.

**Table 6 Predictive performance of top VAGs (and runner ups) in DCM-1 population**

| DCM-1 | | | | | |
| --- | --- | --- | --- | --- | --- |
| VAG combinations* | | | Prevalence, %^1^ | PPV, % (CI)^2^ | Sensitivity, % (CI)^3^ |
| *papX* |  |  | 45 | 96 (90–100) | 54 (44–64) |
| *intS* |  |  | 33 | 95 (87–100) | 38 (29–48) |
| *papE* |  |  | 30 | 94 (86–100) | 35 (26–45) |
| *papD* |  |  | 29 | 94 (85–100) | 34 (25–44) |
| *papGII* |  |  | 49 | 93 (86–98) | 56 (46–66) |
| is3-III |  |  | 54 | 92 (85–98) | 61 (51–70) |
| *papF* |  |  | 52 | 92 (85–98) | 59 (49–68) |
| *iha* |  |  | 44 | 90 (82–98) | 48 (38–58) |
| *kpsT* |  |  | 26 | 90 (79–100) | 29 (20–38) |
| *sat* |  |  | 42 | 90 (81–98) | 46 (37–56) |
| *tnpB* |  |  | 32 | 89 (79–98) | 35 (26–45) |
| *insN* |  |  | 63 | 89 (82–96) | 69 (60–78) |
| is3-IV |  |  | 24 | 89 (76–100) | 26 (17–35) |
| *iucC* |  |  | 31 | 89 (78–98) | 34 (25–44) |
| *tnpJ* |  |  | 39 | 89 (79–98) | 42 (33–52) |
| isl3 |  |  | 53 | 89 (81–96) | 58 (48–67) |
| *iucB* |  |  | 30 | 89 (77–98) | 33 (24–43) |
| *papC* |  |  | 60 | 89 (81–96) | 65 (55–74) |
| *papI* |  |  | 51 | 89 (80–96) | 56 (46–66) |
| *senB* |  |  | 22 | 88 (75–100) | 24 (16–32) |
| *ykfF* | *papX* |  | 39 | 100 (100–100) | 47 (38–57) |
| *papX* | *papC* |  | 36 | 100 (100–100) | 44 (34–54) |
| *papX* | *papF* |  | 34 | 100 (100–100) | 42 (33–52) |
| *papX* | *papH* |  | 34 | 100 (100–100) | 42 (33–52) |
| *papX* | *papI* |  | 34 | 100 (100–100) | 42 (33–52) |
| *papX* | *papGII* |  | 32 | 100 (100–100) | 39 (30–49) |
| int-p4 | *intS* |  | 28 | 100 (100–100) | 34 (25–44) |
| shiA-h | *intS* |  | 28 | 100 (100–100) | 34 (25–44) |
| *papX* | marR |  | 25 | 100 (100–100) | 31 (22–40) |
| *papX* | *insA* |  | 25 | 100 (100–100) | 31 (22–40) |
| *papX* | *papK* |  | 24 | 100 (100–100) | 30 (21–39) |
| *vat* | *papX* |  | 23 | 100 (100–100) | 28 (19–37) |
| *rhuM* | *intS* |  | 23 | 100 (100–100) | 28 (19–37) |
| *papJ* | *papX* |  | 23 | 100 (100–100) | 28 (19–37) |
| *papX* | *intS* |  | 23 | 100 (100–100) | 28 (19–37) |
| *papX* | *papE* |  | 23 | 100 (100–100) | 28 (19–37) |
| *z1216* | *papX* |  | 22 | 100 (100–100) | 27 (18–36) |
| *papX* | *iucA* |  | 22 | 100 (100–100) | 27 (18–36) |
| *papX* | *iucB* |  | 22 | 100 (100–100) | 27 (18–36) |
| is3-III | *intS* |  | 22 | 100 (100–100) | 27 (18–36) |
| *papX* | *fecE* | *fepE* | 34 | 100 (100–100) | 42 (42–42) |
| *papX* | *fecA* | *fepE* | 34 | 100 (100–100) | 42 (42–42) |
| *papX* | *fecB* | *fepE* | 34 | 100 (100–100) | 42 (42–42) |
| *papX* | *fecC* | *fepE* | 34 | 100 (100–100) | 42 (42–42) |
| *papX* | *fecD* | *fepE* | 34 | 100 (100–100) | 42 (42–42) |
| *papX* | *fecI* | *fepE* | 34 | 100 (100–100) | 42 (42–42) |
| *papX* | *fecR* | *fepE* | 34 | 100 (100–100) | 42 (42–42) |
| *istA* | *papX* | *fepE* | 33 | 100 (100–100) | 40 (40–40) |
| *papX* | is3-III | *fepE* | 29 | 100 (100–100) | 36 (36–36) |
| *kpsD* | *fecE* | *intS* | 22 | 100 (100–100) | 27 (27–27) |
| *kpsD* | *fecA* | *intS* | 22 | 100 (100–100) | 27 (27–27) |
| *kpsD* | *fecB* | *intS* | 22 | 100 (100–100) | 27 (27–27) |
| *kpsD* | *fecC* | *intS* | 22 | 100 (100–100) | 27 (27–27) |
| *kpsD* | *fecD* | *intS* | 22 | 100 (100–100) | 27 (27–27) |
| *kpsD* | *fecI* | *intS* | 22 | 100 (100–100) | 27 (27–27) |
| *kpsD* | *fecR* | *intS* | 22 | 100 (100–100) | 27 (27–27) |
| *kpsM* | *fecE* | *intS* | 22 | 100 (100–100) | 27 (27–27) |
| *kpsM* | *fecA* | *intS* | 22 | 100 (100–100) | 27 (27–27) |
| *kpsM* | *fecB* | *intS* | 22 | 100 (100–100) | 27 (27–27) |
| *kpsM* | *fecC* | *intS* | 22 | 100 (100–100) | 27 (27–27) |

Table 6**:** *E. coli* bacteremia isolates from DCM-1 population (n = 119) virulence associated genes as singles, pairs, and triplets for predicting UTI as source of bacteremia. Top 20 results each, sorted for sensitivity >20% and by highest PPV. Top performing 20 pairs removed from triplet calculation. *^1^Prevalence:* The proportion (%) of isolates with the specific VAG or VAG combination. *^2^PPV:* The proportion (%) of cases with the specific VAG or VAG combination associated with UTI as source of bacteremia. *^3^Sensitivity:* The proportion (%) of cases with UTI as source of bacteremia correctly predicted with the specific VAG or VAG combination. Abbreviations: VAG, virulence associated gene; CI, confidence interval; PPV, positive predictive value; DCM, Department of Clinical Microbiology; UTI, urinary tract infection.

*Virulence associated gene descriptions and representative sequences are found in Table 4 in Supplementary Appendix.

**Table 7 Predictive performance of top VAGs (and runner ups) in DCM-2 population**

| DCM-2 | | | | | |
| --- | --- | --- | --- | --- | --- |
| VAG combinations* | | | Prevalence, %^1^ | PPV, % (CI)^2^ | Sensitivity, % (CI)^3^ |
| *kpsT* |  |  | 23 | 67 (47–85) | 31 (19–44) |
| *fimC4* |  |  | 20 | 67 (45–86) | 27 (15–40) |
| *fimD* |  |  | 20 | 67 (45–86) | 27 (15–40) |
| *fimA* |  |  | 21 | 64 (43–83) | 27 (15–40) |
| *vat* |  |  | 26 | 63 (44–81) | 33 (20–46) |
| *sat* |  |  | 25 | 62 (42–80) | 31 (19–44) |
| *tnaC* |  |  | 31 | 61 (43–77) | 38 (25–52) |
| *intS* |  |  | 29 | 60 (42–77) | 35 (22–48) |
| *iucB* |  |  | 24 | 60 (40–79) | 29 (17–42) |
| *hemR* |  |  | 45 | 60 (45–73) | 54 (40–67) |
| *iucC* |  |  | 26 | 59 (40–78) | 31 (19–44) |
| *hlyA* |  |  | 21 | 59 (38–80) | 25 (14–37) |
| *senB* |  |  | 21 | 59 (38–80) | 25 (14–37) |
| *insF* |  |  | 37 | 59 (43–74) | 44 (31–58) |
| *insA* |  |  | 32 | 59 (42–75) | 38 (25–52) |
| *papC* |  |  | 32 | 59 (42–75) | 38 (25–52) |
| *iha* |  |  | 28 | 59 (40–76) | 33 (20–46) |
| is3-II |  |  | 41 | 58 (43–73) | 48 (35–62) |
| *papI* |  |  | 30 | 58 (40–75) | 35 (22–48) |
| is4-I |  |  | 25 | 58 (38–77) | 29 (17–42) |
| *istA* | *insF* |  | 20 | 76 (57–94) | 31 (19–44) |
| *kpsM* | *insF* |  | 21 | 73 (53–90) | 31 (19–44) |
| *kpsT* | gtpp |  | 21 | 73 (53–90) | 31 (19–44) |
| *insF* | hypp |  | 24 | 72 (53–89) | 35 (22–48) |
| *ykfF* | *insF* |  | 20 | 71 (52–88) | 33 (20–46) |
| *kpsT* | *yeeV* |  | 20 | 71 (50–90) | 29 (17–42) |
| *ykfF* | *tnaC* |  | 20 | 71 (50–90) | 29 (17–42) |
| *insF* | *tnpB* |  | 20 | 71 (50–90) | 29 (17–42) |
| *hemR* | *ag43* |  | 20 | 71 (50–90) | 29 (17–42) |
| *tnaC* | *fimB* |  | 23 | 71 (50–90) | 29 (17–42) |
| *kpsD* | *insF* |  | 22 | 70 (50–88) | 31 (19–44) |
| *kpsT* | *rox* |  | 22 | 70 (50–88) | 31 (19–44) |
| *ykfF* | is3-II |  | 22 | 70 (50–88) | 31 (19–44) |
| *kpsD* | *vat* |  | 21 | 68 (48–86) | 33 (20–46) |
| *sat* | is3-II |  | 21 | 68 (47–87) | 29 (17–42) |
| *kpsT* | trpos |  | 21 | 68 (48–87) | 29 (17–42) |
| *z1203* | *vat* |  | 21 | 68 (48–88) | 29 (17–42) |
| *z1204* | *vat* |  | 21 | 68 (47–88) | 29 (17–42) |
| *ykfF* | *vat* |  | 21 | 68 (48–88) | 29 (17–42) |
| is3-II | is4-II |  | 24 | 68 (47–88) | 29 (17–42) |
| *kpsM* | *z1226* | *hemR* | 22 | 78 (61–93) | 40 (27–54) |
| *z1226* | is3-I | *chuY* | 22 | 78 (60–95) | 35 (22–48) |
| *z1226* | is3-I | *chuA* | 22 | 78 (60–94) | 35 (22–48) |
| *z1226* | is3-I | *chuT* | 22 | 78 (60–94) | 35 (22–48) |
| *z1226* | is3-I | *chuS* | 22 | 78 (60–95) | 35 (22–48) |
| *z1226* | is3-I | *chuW* | 22 | 78 (60–95) | 35 (22–48) |
| *z1226* | is3-I | *chuU* | 26 | 78 (60–94) | 35 (22–48) |
| *kpsD* | *hemR* | *fecE* | 21 | 77 (58–94) | 33 (20–46) |
| *kpsD* | *hemR* | *fecA* | 21 | 77 (58–94) | 33 (20–46) |
| *kpsD* | *hemR* | *fecB* | 21 | 77 (58–94) | 33 (20–46) |
| *kpsD* | *hemR* | *fecC* | 21 | 77 (58–94) | 33 (20–46) |
| *kpsD* | *hemR* | *fecD* | 21 | 77 (58–94) | 33 (20–46) |
| *kpsD* | *hemR* | *fecI* | 21 | 77 (58–94) | 33 (20–46) |
| *kpsD* | *hemR* | *fecR* | 21 | 77 (58–94) | 33 (20–46) |
| *kpsM* | *insN* | *hemR* | 21 | 77 (58–94) | 33 (20–46) |
| *kpsM* | *hemR* | *fecE* | 21 | 77 (58–94) | 33 (20–46) |
| *kpsM* | *hemR* | *fecA* | 21 | 77 (58–94) | 33 (20–46) |
| *kpsM* | *hemR* | *fecB* | 21 | 77 (58–94) | 33 (20–46) |
| *kpsM* | *hemR* | *fecC* | 21 | 77 (58–94) | 33 (20–46) |
| *kpsM* | *hemR* | *fecD* | 21 | 77 (58–94) | 33 (20–46) |

Table 7: *E. coli* bacteremia isolates from DCM-2 population (n = 105) virulence associated genes as singles, pairs, and triplets for predicting UTI as source of bacteremia. Top 20 results each, sorted for sensitivity >20% and by highest PPV. Top performing 20 pairs removed from triplet calculation. *^1^Prevalence:* The proportion (%) of isolates with the specific VAG or VAG combination. *^2^PPV:* The proportion (%) of cases with the specific VAG or VAG combination associated with UTI as source of bacteremia. *^3^Sensitivity:* The proportion (%) of cases with UTI as source of bacteremia correctly predicted with the specific VAG or VAG combination. Abbreviations: VAG, virulence associated gene; CI, confidence interval; PPV, positive predictive value; DCM, Department of Clinical Microbiology; UTI, urinary tract infection.

*Virulence associated gene descriptions and representative sequences are found in Table 4 in Supplementary Appendix.

**Table 8 Predictive performance of top VAGs across different hospital populations**

| DCM-1 | | | | | |
| --- | --- | --- | --- | --- | --- |
| VAG combinations* | | | Prevalence, %*^1^* | PPV, % (CI)*^2^* | Sensitivity, % (CI)*^3^* |
| *kpsT* |  |  | 30 | 90 (47–85) | 29 (19–44) |
| *fimA* |  |  | 33 | 86 (42–83) | 31 (15–40) |
| *fimC4* |  |  | 32 | 85 (45–86) | 30 (15–39) |
| *fimD* |  |  | 31 | 85 (45–86) | 29 (15–39) |
| *vat* |  |  | 49 | 84 (44–81) | 44 (20–46) |
| *kpsT* | gtpp |  | 29 | 90 (78–100) | 28 (19–37) |
| *kpsT* | *yeeV* |  | 23 | 88 (73–100) | 22 (14–30) |
| *kpsM* | *insF* |  | 30 | 84 (70–96) | 27 (18–36) |
| *istA* | *insF* |  | 29 | 83 (69–96) | 26 (17–35) |
| *insF* | hypp |  | 29 | 83 (69–96) | 26 (17–35) |
| *z1226* | is3-I | *chuY* | 45 | 81 (69–91) | 39 (30–49) |
| *z1226* | is3-I | *chuA* | 45 | 81 (69–91) | 39 (30–49) |
| *z1226* | is3-I | *chuS* | 45 | 81 (69–91) | 39 (30–49) |
| *z1226* | is3-I | *chuW* | 45 | 81 (69–91) | 39 (30–49) |
| *z1226* | is3-I | *chuT* | 45 | 81 (69–91) | 39 (29–49) |
| DCM-2 | | | | | |
| VAG combinations | | | Prevalence, %*^1^* | PPV, % (CI)*^2^* | Sensitivity, % (CI)*^3^* |
| *papD* |  |  | 16 | 71 (47–92) | 23 (12–35) |
| *papGII* |  |  | 18 | 68 (46–89) | 25 (14–37) |
| *intS* |  |  | 29 | 60 (42–77) | 35 (22–48) |
| *papX* |  |  | 32 | 47 (30–64) | 31 (18–44) |
| *papE* |  |  | 17 | 39 (17–62) | 13 (5–23) |
| *papX* | *papF* |  | 12 | 69 (42–93) | 17 (8–28) |
| *papX* | *papC* |  | 17 | 67 (43–88) | 23 (12–35) |
| *papX* | *papI* |  | 16 | 65 (40–88) | 21 (11–33) |
| *papX* | *papH* |  | 15 | 62 (38–86) | 19 (9–31) |
| *ykfF* | *papX* |  | 22 | 52 (31–73) | 23 (12–35) |
| *papX* | *fecE* | *fepE* | 17 | 50 (27–74) | 17 (8–28) |
| *papX* | *fecA* | *fepE* | 17 | 50 (26–74) | 17 (8–28) |
| *papX* | *fecB* | *fepE* | 17 | 50 (26–74) | 17 (8–28) |
| *papX* | *fecC* | *fepE* | 17 | 50 (26–74) | 17 (8–28) |
| *papX* | *fecD* | *fepE* | 17 | 50 (27–74) | 17 (8–28) |

Table 8: Displaying the five *E. coli* VAG singles, pairs, and triplets that best predict UTI as source of bacteremia after swapping DCM population and recalculating. Each DCM populations high-performing VAGs were rerun on the other DCM’s population data. Sorted to display highest PPV within each group. *^1^Prevalence:* The proportion (%) of isolates with the specific VAG or VAG combination. *^2^PPV:* The proportion (%) of cases with the specific VAG or VAG combination associated with UTI as source of bacteremia. *^3^Sensitivity:* The proportion (%) of cases with UTI as source of bacteremia correctly predicted with the specific VAG or VAG combination. Abbreviations: VAG, virulence associated gene; CI, confidence interval; PPV, positive predictive value; DCM, Department of Clinical Microbiology; UTI, urinary tract infection.

*Virulence associated gene descriptions and representative sequences are found in Table 4 in Supplementary Appendix.

**Table 9 MLST and cgMLST distribution by DCM and source of bacteremia**

| DCM-1 | | | |  | DCM-2 | | | |
| --- | --- | --- | --- | --- | --- | --- | --- | --- |
| UTI source  of bacteremia | | non-UTI source  of bacteremia | |  | UTI source  of bacteremia | | non-UTI source  of bacteremia | |
| ST Warwick (Complex Type) | n | ST Warwick (Complex Type) | n |  | ST Warwick (Complex Type) | n | ST Warwick (Complex Type) | n |
| **73** | **17** | **73** | **5** |  | **131** | **6** | **131** | **8** |
| 6646 | 1 | 6648 | 1 |  | 1168 | 1 | 1260 | 1 |
| 6653 | 1 | 6660 | 1 |  | 5325 | 1 | 5368 | 1 |
| 6673 | 1 | 6693 | 1 |  | 5450 | 1 | 5480 | 1 |
| 6675 | 2 | 6699 | 1 |  | 5515 | 2 | 6894 | 1 |
| 6683 | 1 | 6748 | 1 |  | 6865 | 1 | 6897 | 1 |
| 6700 | 1 | **131** | **3** |  | **69** | **4** | 6914 | 1 |
| 6701 | 1 | 5619 | 1 |  | 6876 | 1 | 6920 | 1 |
| 6711 | 1 | 6678 | 1 |  | 6884 | 1 | 6941 | 1 |
| 6714 | 1 | 6742 | 1 |  | 6885 | 1 | **69** | **6** |
| 6717 | 1 | **348** | **2** |  | 6931 | 1 | 6617 | 1 |
| 6726 | 1 | 3024 | 2 |  | **95** | **3** | 6864 | 1 |
| 6733 | 1 |  |  |  | 6926 | 1 | 6882 | 1 |
| 6734 | 1 |  |  |  | 6942 | 1 | 6909 | 1 |
| 6743 | 1 |  |  |  | 6946 | 1 | 6912 | 1 |
| 6747 | 1 |  |  |  |  |  | 6924 | 1 |
| 6750 | 1 |  |  |  |  |  | **95** | **4** |
| **95** | **17** |  |  |  |  |  | 5424 | 1 |
| 6575 | 1 |  |  |  |  |  | 6797 | 1 |
| 6643 | 1 |  |  |  |  |  | 6902 | 1 |
| 6676 | 1 |  |  |  |  |  | 6923 | 1 |
| 6684 | 1 |  |  |  |  |  |  |  |
| 6686 | 1 |  |  |  |  |  |  |  |
| 6698 | 1 |  |  |  |  |  |  |  |
| 6704 | 1 |  |  |  |  |  |  |  |
| 6710 | 1 |  |  |  |  |  |  |  |
| 6713 | 1 |  |  |  |  |  |  |  |
| 6721 | 1 |  |  |  |  |  |  |  |
| 6722 | 1 |  |  |  |  |  |  |  |
| 6723 | 1 |  |  |  |  |  |  |  |
| 6728 | 1 |  |  |  |  |  |  |  |
| 6731 | 1 |  |  |  |  |  |  |  |
| 6735 | 1 |  |  |  |  |  |  |  |
| 6746 | 1 |  |  |  |  |  |  |  |
| 6749 | 1 |  |  |  |  |  |  |  |
| **131** | **16** |  |  |  |  |  |  |  |
| 1260 | 1 |  |  |  |  |  |  |  |
| 533 | 1 |  |  |  |  |  |  |  |
| 5355 | 2 |  |  |  |  |  |  |  |
| 5362 | 1 |  |  |  |  |  |  |  |
| 5365 | 2 |  |  |  |  |  |  |  |
| 5529 | 1 |  |  |  |  |  |  |  |
| 5629 | 1 |  |  |  |  |  |  |  |
| 6645 | 1 |  |  |  |  |  |  |  |
| 6680 | 1 |  |  |  |  |  |  |  |
| 6702 | 1 |  |  |  |  |  |  |  |
| 6703 | 1 |  |  |  |  |  |  |  |
| 6705 | 1 |  |  |  |  |  |  |  |
| 6720 | 1 |  |  |  |  |  |  |  |
| 6724 | 1 |  |  |  |  |  |  |  |

Table 9: Displaying the top 3 ST within each type of bacteremia source in each DCM. Abbreviations: MLST, multi-locus sequence typing; cgMLST, core genome multi-locus sequence typing; ST, sequence typing; DCM, Department of Clinical Microbiology; UTI, urinary tract infection.
